# The intrinsically disordered TSSC4 protein acts as a helicase inhibitor, placeholder and multi-interaction coordinator during snRNP assembly and recycling

**DOI:** 10.1093/nar/gkac087

**Published:** 2022-02-21

**Authors:** Alexandra Bergfort, Tarek Hilal, Benno Kuropka, İbrahim Avşar Ilik, Gert Weber, Tuğçe Aktaş, Christian Freund, Markus C Wahl

**Affiliations:** Freie Universität Berlin, Institute of Chemistry and Biochemistry, Laboratory of Structural Biochemistry, Takustr. 6, D-14195 Berlin, Germany; Freie Universität Berlin, Institute of Chemistry and Biochemistry, Laboratory of Structural Biochemistry, Takustr. 6, D-14195 Berlin, Germany; Freie Universität Berlin, Institute of Chemistry and Biochemistry, Research Center of Electron Microscopy, Fabeckstr. 36a, 14195 Berlin, Germany; Freie Universität Berlin, Institute of Chemistry and Biochemistry, Laboratory of Protein Biochemistry, Thielallee 63, D-14195, Berlin, Germany; Freie Universität Berlin, Institute of Chemistry and Biochemistry, Core Facility BioSupraMol, Thielallee 63, D-14195, Berlin, Germany; Max Planck Institute for Molecular Genetics, Ihnestr. 63, D-14195 Berlin, Germany; Helmholtz-Zentrum Berlin für Materialien und Energie, Macromolecular Crystallography, Albert-Einstein-Str. 15, D-12489 Berlin, Germany; Max Planck Institute for Molecular Genetics, Ihnestr. 63, D-14195 Berlin, Germany; Freie Universität Berlin, Institute of Chemistry and Biochemistry, Laboratory of Protein Biochemistry, Thielallee 63, D-14195, Berlin, Germany; Freie Universität Berlin, Institute of Chemistry and Biochemistry, Laboratory of Structural Biochemistry, Takustr. 6, D-14195 Berlin, Germany; Helmholtz-Zentrum Berlin für Materialien und Energie, Macromolecular Crystallography, Albert-Einstein-Str. 15, D-12489 Berlin, Germany

## Abstract

Biogenesis of spliceosomal small nuclear ribonucleoproteins (snRNPs) and their recycling after splicing require numerous assembly/recycling factors whose modes of action are often poorly understood. The intrinsically disordered TSSC4 protein has been identified as a nuclear-localized U5 snRNP and U4/U6-U5 tri-snRNP assembly/recycling factor, but how TSSC4’s intrinsic disorder supports TSSC4 functions remains unknown. Using diverse interaction assays and cryogenic electron microscopy-based structural analysis, we show that TSSC4 employs four conserved, non-contiguous regions to bind the PRPF8 Jab1/MPN domain and the SNRNP200 helicase at functionally important sites. It thereby inhibits SNRNP200 helicase activity, spatially aligns the proteins, coordinates formation of a U5 sub-module and transiently blocks premature interaction of SNRNP200 with at least three other spliceosomal factors. Guided by the structure, we designed a TSSC4 variant that lacks stable binding to the PRPF8 Jab1/MPN domain or SNRNP200 *in vitro*. Comparative immunoprecipitation/mass spectrometry from HEK293 nuclear extract revealed distinct interaction profiles of wild type TSSC4 and the variant deficient in PRPF8/SNRNP200 binding with snRNP proteins, other spliceosomal proteins as well as snRNP assembly/recycling factors and chaperones. Our findings elucidate molecular strategies employed by an intrinsically disordered protein to promote snRNP assembly, and suggest multiple TSSC4-dependent stages during snRNP assembly/recycling.

## Graphical Abstract

**Graphical Abstract:**
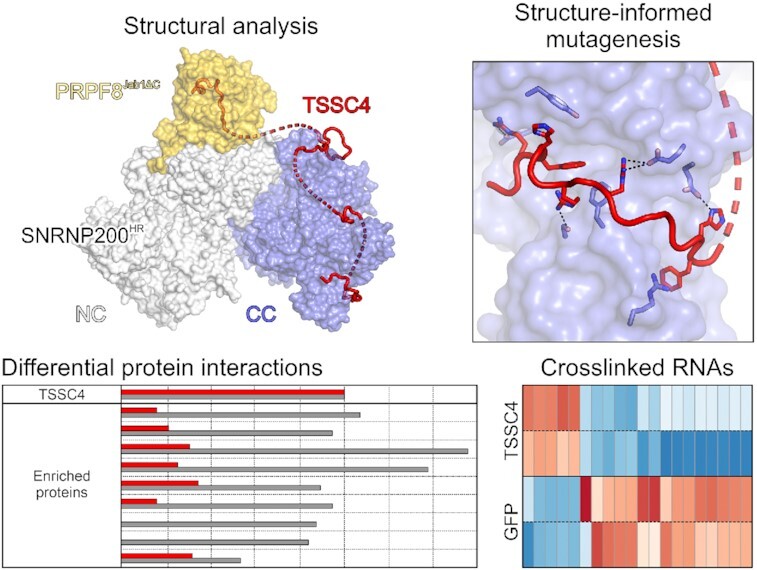
Guided by a cryoEM structure of assembly chaperone TSSC4 in complex with portions of SNRNP200 and PRPF8, Bergfort *et al.* investigated how the TSSC4 intrinsic disorder supports snRNP assembly and recycling.

## INTRODUCTION

Precursor messenger RNA (pre-mRNA) splicing, the process by which non-coding introns are excised and coding exons are ligated, is carried out by a large and dynamic ribonucleoprotein (RNP) machinery, the spliceosome (1–4). The U1, U2, U4, U5 and U6 small nuclear (sn) RNPs constitute the main building blocks of the major spliceosome. A corresponding set of U11, U12, U4atac, U5 and U6atac snRNPs participates in formation of the minor spliceosome (5,6). All snRNPs contain a particle-specific, uridine-rich snRNA, seven common Sm proteins (or like-Sm [LSm] proteins in the case of U6 and U6atac) and a variable number of particle-specific proteins (7).

Spliceosomal snRNPs are assembled *via* complex pathways that require a large number of assembly factors and chaperones. Apart from U6 and U6atac snRNAs, which are transcribed by RNA polymerase III, all other snRNAs are generated by RNA polymerase II (8,9). While U6 and U6atac snRNAs were thought to mature and assemble snRNPs in the nucleus (10), recent analyses suggested that maturation/assembly of all snRNAs/snRNPs involve nuclear-cytoplasmic shuttling (11). The RNA polymerase II-transcribed snRNAs undergo co-transcriptional m^7^G capping, which recruits the cap-binding complex and additional factors, leading to nuclear export of the snRNAs (8,9,12). In the cytoplasm, a heptameric ring of Sm proteins binds the snRNAs, facilitated by the protein arginine methyltransferase 5 (PRMT5) and the survival motor neuron (SMN) complexes (8,9,12,13). The resulting Sm core particles recruit tri-methyl-guanosine synthase 1, leading to hyper-methylation of the m^7^G cap to a m^2,2,7^G cap and subsequent nuclear import, mediated by snurportin, importin-β and the SMN complex (8,9,12). Final maturation of snRNPs in the nucleus, including incorporation of snRNP-specific proteins, takes place in Cajal bodies (12,14,15).

Some snRNPs are profoundly remodeled during pre-mRNA splicing. For instance, the U5 snRNP 200 kDa helicase (SNRNP200; Brr2p in yeast) unwinds the initially extensively base-paired U4 and U6 snRNAs during spliceosome activation and releases U4 snRNA and all U4/U6-associated proteins (16), necessitating re-assembly of the U4/U6 di-snRNP and the U4/U6-U5 tri-snRNP after splicing. As another example in humans, the 20S U5 snRNP associates with the pre-mRNA-processing factor (PRPF) 19 complex and additional factors during splicing, ultimately being released as a 35S particle (17), from which the U5 snRNP has to be regenerated. Like *de novo* snRNP biogenesis, recycling of snRNPs after splicing requires specialized molecular machinery and is also thought to take place in Cajal bodies (14,15,18,19).

While the molecular mechanisms underlying assembly of Sm core RNPs in the cytoplasm have been studied in detail (8,9,12), much less is known about later stages of snRNP biogenesis and snRNP recycling in the nucleus. Several proteins have by now been implicated in these latter processes. For instance, the spliceosome-associated factor 3 (SART3) acts as a U4/U6 assembly and recycling factor that targets U6 snRNP to Cajal bodies (20,21). A1 cistron-splicing factor (AAR2), whose Aar2p ortholog has been characterized as a U5 snRNP assembly and recycling factor in yeast (22–25), appears to also interact with U5 snRNP-specific proteins in human (26,27). The HSP90/R2TP chaperone system has been proposed to promote assembly of a U5 snRNP sub-module composed of the U5 proteins PRPF8, SNRNP200, 116 kDa U5 small nuclear ribonucleoprotein component (EFTUD2; Snu114p in yeast) and SNRNP40 (26,28). This tetrameric sub-module had previously been shown to form a stable building block of the human U5 snRNP (29). The same chaperone system, together with the adaptor protein, NUFIP, was shown to interact with the SMN complex to mediate assembly of U4 snRNP-specific proteins (30).

Recently, the tumor-suppressing sub-chromosomal transferable fragment candidate 4 protein (TSSC4) has been characterized as an additional factor involved in U5 snRNP assembly and recycling, and was proposed to also facilitate U4/U6-U5 tri-snRNP formation (31). It was found to exist in complexes with several U5 snRNP components, in particular PRPF8, SNRNP200, EFTUD2 and U5 snRNA, as well as PRPF19 complex proteins, dependent in part on different regions in TSSC4 (31). SiRNA-mediated knock-down of TSSC4 led to accumulation of U4, U5 and U6 snRNAs as well as of several U5-specific proteins in Cajal bodies, and resulted in reduced levels of U4/U6-U5 tri-snRNP in favor of free U5 snRNP (31). However, how TSSC4 exerts snRNP assembly/recycling functions on a molecular level, in particular in light of its predicted intrinsic disorder (31), remains unknown.

Here, we show that TSSC4 directly interacts with the SNRNP200 RNA helicase and the PRPF8 spliceosomal scaffold, and present a high-resolution cryogenic electron microscopy (cryoEM) structure of TSSC4 in complex with the helicase region of SNRNP200 and the Jab1/MPN (Jab1) domain of PRPF8. Based on the structure, we designed a TSSC4 variant that lacks stable binding to SNRNP200 or the PRPF8 Jab1 domain and employed it in comparative protein and RNA interactome studies. Our results reveal how, due to its intrinsic disorder, TSSC4 can occupy multiple, distant binding sites on PRPF8 and SNRNP200, resorting to short, discontinuous and conserved binding regions. We delineate PRPF8/SNRNP200-dependent and independent interactions of TSSC4 with additional spliceosomal proteins, snRNP assembly factors, chaperones and snRNAs. Our results reveal how TSSC4 aids snRNP assembly/recycling by suppressing SNRNP200 RNA helicase activity, spatially organizing U5 snRNP proteins, guiding formation of the PRPF8-SNRNP200-EFTUD2-SNRNP40 U5 sub-module, intermittently blocking alternative interactions of SNRNP200 and coordinating interactions with other snRNP assembly factors and chaperones.

## MATERIALS AND METHODS

### Protein production and reconstitution of protein complexes

A DNA fragment encoding human TSSC4 was cloned into the pETM30 vector (EMBL, Heidelberg) *via Nco*I and *Xho*I restriction sites to drive production of an N-terminally His_6_-GST-tagged protein (TEV-cleavable; GST-TSSC4). Plasmids for the production of TSSC4 variants were obtained by site-directed mutagenesis using Q5 High-Fidelity Polymerase (NEB).

SNRNP200 variants and variants of the PRPF8 Jab1/MPN domain, lacking (residues 2064–2320; PRPF8^Jab1ΔC^) or containing (residues 2064–2335; PRPF8^Jab1^) a SNRNP200-inhibitory C-terminal tail, were produced *via* recombinant baculoviruses in Hi-5 insect cells and purified as described before (32,33). FBP21 and C9ORF78 variants were produced in *Escherichia coli* BL21-CodonPlus (DE3)-RIL cells (Agilent) and purified as described before (34,35). TSSC4 variants were produced in *E. coli* BL21-CodonPlus (DE3)-RIL cells cultivated in auto-inducing medium (36) for 4 h at 37°C, followed by 72 h at 18°C. Cells were harvested by centrifugation and re-suspended in lysis buffer (50 mM Tris–HCl, pH 8.0, 200 mM NaCl, 1 mM DTT) supplemented with protease inhibitors (Roche) and DNase I (Roche; 2 mg/50 ml) and lysed by sonication. The lysate was cleared by centrifugation, and proteins were ammonium sulfate-precipitated (80% saturation) to eliminate nucleic acid contamination. The protein pellet was dissolved in lysis buffer, the target protein was captured on Glutathione Sepharose 4 Fast Flow resin (Cytiva), washed with 50 ml lysis buffer, followed by 50 ml 50 mM Tris–HCl, pH 8.0, 10 mM ATP, 10 mM MgCl_2_, 150 mM KCl, followed by 50 ml lysis buffer, and eluted with 35 ml lysis buffer supplemented with 10 mM reduced glutathione. Fractions containing the proteins of interest were concentrated to 15 mg/ml by ultrafiltration (Amicon) and subjected to size exclusion chromatography (SEC) on a 16/60 Superdex 200 column (Cytiva) with 20 mM Tris–HCl, pH 8.0, 200 mM NaCl, 1 mM DTT.

For reconstitution of a complex comprising the SNRNP200 helicase region (residues 395–2129; SNRNP200^HR^) and TSSC4, individually produced His-SNRNP200^HR^, containing an TEV-cleavable N-terminal His_10_-tag (cleared lysate), and GST-TSSC4 (dissolved ammonium sulfate-precipitated fraction) were mixed and incubated for 1 h at 4°C. The complex was captured on and eluted from Glutathione Sepharose 4 Fast Flow resin as described above, followed by addition of TEV protease (1:20 TEV:target complex mass ratio) and overnight dialysis against 20 mM Tris–HCl, pH 8.0, 200 mM NaCl, 1 mM DTT at 4°C. After tag cleavage, the complex was subjected to SEC on a 16/60 Superdex 200 column with 20 mM Tris–HCl, pH 8.0, 200 mM NaCl, 1 mM DTT. Fractions of interest were pooled, concentrated to 5.7 mg/ml by ultrafiltration, aliquoted, flash-frozen in liquid nitrogen and stored at -80°C.

For structural analysis *via* cryogenic electron microscopy (cryoEM), 1.8 nmol of the SNRNP200^HR^-TSSC4 complex were incubated with a 1.5-fold molar excess of PRPF8^Jab1ΔC^ for 10 min on ice. The SNRNP200^HR^–PRPF8^Jab1ΔC^–TSSC4 complex was purified by SEC on a Superdex 200 increase 3.2/300 column (Cytiva) with 20 mM Tris–HCl, pH 8.0, 150 mM NaCl, 1 mM DTT. Fractions of interest were concentrated to 5 mg/ml using ultrafiltration and used immediately for grid preparation.

### Analytical size exclusion chromatography

75 μg of an SNRNP200 variant (SNRNP200^FL^, SNRNP200^HR^, SNRNP200^NC^ or SNRNP200^CC^) and a 1.5-fold molar excess (with respect to the SNRNP200 variant) of a GST-TSSC4 variant or GST, or 30 μg of a GST-TSSC4 variant and an equimolar amount of PRPF8^Jab1ΔC^, were mixed in 50 μl volumes. Individual proteins or protein mixtures were pre-incubated on ice for 10 min and subjected to analytical SEC on a Superdex 200 increase 3.2/300 column with 20 mM Tris–HCl, pH 8.0, 200 mM NaCl, 1 mM DTT. 60 μl elution fractions were collected, of which 15 μl were analyzed by SDS-PAGE. For competitive binding assays, a C-terminal fragment of FBP21 (residues 200–376; FBP21^200–376^) was employed. 5 μM SNRNP200^HR^-FBP21^200–376^ complex or SNRNP200^HR^-C9ORF78 complex were incubated with 5 μM of GST-TSSC4 in 50 μl volumes for 10 min on ice, followed by analytical SEC as described above.

### Limited proteolysis

A 50 μl mixture of 16 μM SNRNP200^HR^ and 16 μM GST-TSSC4 in 10 mM Tris–HCl, pH 8.0, 200 mM NaCl, 1 mM DTT was incubated for 5 min on ice. 0.12 μg of chymotrypsin (Roche) were added and the mixture was incubated for 45 min at room temperature. Reaction products were analyzed *via* analytical SEC on a Superdex increase 3.2/300 column with 10 mM Tris–HCl, pH 8.0, 200 mM NaCl as described above. Bands of interest were excised, in-gel digested with trypsin using a standard protocol (37), extracted, dried and analyzed by matrix-assisted laser desorption ionization-time of flight mass spectrometry (MALDI-TOF-MS).

### Peptide SPOT array

Membranes with spots of 25-residue peptides of TSSC4 with an overlap of 20 residues were obtained from Dr. Rudolf Volkmer, Charite – Universitätsmedizin Berlin. Membranes were pre-washed once with 100% ethanol and three times with phosphate buffered saline (PBS) supplemented with 1 mM DTT. The remaining binding capacity of the membranes was blocked by a 3-h incubation with blocking buffer (5% [w/v] BSA in PBS supplemented with 1 mM DTT). Subsequently, the membranes were incubated overnight at 4°C with N-terminally His_10_-tagged full-length (FL) SNRNP200 (His-SNRNP200), His-SNRNP200^HR^, N-terminally His_10_-tagged SNRNP200 N-terminal helicase cassette (residues 395–1324; His-SNRNP200^NC^), N-terminally His_10_-tagged SNRNP200 C-terminal helicase cassette (residues 1282–2136; His-SNRNP200^CC^), at a concentration of or 60 μg/ml (His-SNRNP200^FL^), 50 μg/ml (His-SNRNP200^HR^) or 25 μg/ml (His-SNRNP200^NC^, His-SNRNP200^CC^) in binding buffer (10 mM Tris–HCl, pH 7.5, 200 mM NaCl, 2 mM DTT). As a negative control, one membrane was incubated with binding buffer without added protein. The membranes where then washed three times with PBS supplemented with 0.05% (v/v) Tween20, 1 mM DTT (PBST) and incubated with a horse radish peroxidase (HRP)-coupled anti-His antibody (Miltenyi Biotech) diluted 1:5000 in PBS with 5% (w/v) BSA for 1 h at room temperature. After washing the membranes three times in PBST, the peptide SPOT arrays were developed with HRP juice (p.j.k. GmbH) and imaged on an Intas Advanced Fluorescence and ECL imager.

### U4/U6 snRNA unwinding assays

Production, purification and radioactive labeling of yeast U4 snRNA and U6 snRNA, as well as assembly of yeast U4/U6 di-snRNA (commonly employed in unwinding assays with human SNRNP200), were carried out as described before (38). All U4/U6 unwinding assays were performed at 30°C. Unwinding of 0.6–1.5 nM radioactive U4/U6 di-snRNA were conducted with 100 nM SNRNP200 (FL or HR), 100 nM SNRNP200 (FL or HR) plus 150 nM GST-TSSC4 or 100 nM SNRNP200 (FL or HR) plus 150 nM GST-TSSC4 plus 250 nM PRPF8^Jab1^ or PRPF8^Jab1ΔC^. All reactions were pre-incubated for 3 min in 40 mM Tris–HCl, pH 7.5, 50 mM NaCl, 0.5 mM MgCl_2_, 8% (v/v) glycerol, 15 ng/μl acetylated BSA, 1 U/μl RNase inhibitor, 1.5 mM DTT in a total volume of 120 μl. The unwinding was initiated by addition of 1.7 mM ATP/MgCl_2_. 10 μl samples were withdrawn at the indicated time points and mixed with 10 μl of 40 mM Tris–HCl, pH 7.4, 50 mM NaCl, 25 mM EDTA, 1% (w/v) SDS, 10% (v/v) glycerol, 0.05% (w/v) xylene cyanol, 0.05% (w/v) bromophenol blue to stop the reaction. The samples were run on a 6% non-denaturing PAGE gel for 1 h at 200 V and 4°C. RNA bands were visualized by autoradiography using a phosphoimager and quantified using the Image Quant 5.2 software (Cytiva). Data were fit to a single exponential equation (fraction unwound = A [1 – exp(-*k_u_*t)]); A, amplitude of the reaction; *k_u_*, apparent first-order rate constant of unwinding; *t*, time) using Prism software (GraphPad).

### Cryogenic electron microscopy and single-particle analysis

Freshly prepared SNRNP200^HR^–PRPF8^JabΔC^–TSSC4 complex (5 mg/ml in 20 mM Tris–HCl, pH 8.0, 150 mM NaCl, 1 mM DTT) was supplemented with 0.15% (w/v) *n*-octylglucoside to overcome preferred particle orientation. 3.8 μl of the final mixture were applied to plasma-treated R1.2/1.3 holey carbon grids (Quantifoil Micro Tools GmbH). Grids were plunged into liquid ethane after blotting using a Vitrobot Mark IV device (FEI) at 10°C and 100% humidity. Images were acquired on a FEI Titan Krios G3i (300 kV) with a Falcon 3EC camera operated in counting mode using EPU software (Thermo Fisher Scientific). The dataset was acquired with a nominal magnification of 120,000, resulting in a calibrated pixel size of 0.657 Å/px. A total electron flux of 40 e^–^/Å^2^ was accumulated over an exposure time of 30.58 s.

All image analysis steps were done with cryoSPARC (39). Movie alignment was done with patch motion correction, CTF estimation was conducted by Patch CTF. Class averages of manually selected particle images were used to generate an initial template for reference-based particle picking from 3,349 micrographs. 1,025,529 particle images were extracted with a box size of 384 px and were Fourier-cropped to 64 px for initial analysis. Reference-free 2D classification was used to select 611,449 particle images for further analysis. *Ab initio* reconstruction using a small subset of particles was conducted to generate an initial 3D reference for consecutive iterations of 3D heterogeneous refinement. 397,619 particle images were re-extracted with a box of 432 px, Fourier-cropped to 144 px (1.971 Å/px) and subjected to non-uniform refinement followed by heterogeneous refinement. Finally, 387,973 particle images were re-extracted using local motion correction at full spatial resolution (box size 432 px, 0.657 Å/px) and down-sampled to a box size of 324 px (0.876 Å/px). Non-uniform refinement was applied to generate the final reconstruction at a resolution of 3.05 Å. To improve the density for TSSC4 and aid modeling, local refinement using a mask covering SNRNP200^CC^, PRPF8^Jab1ΔC^ and TSSC4 was conducted, yielding a reconstruction at 2.85 Å resolution.

### Model building and refinement

Model building was carried out in Coot (40). A model of a human SNRNP200^HR^–PRPF8^Jab1ΔC^ complex (PDB ID 6S8Q) (41) was placed into the final cryoEM reconstruction before local refinement using PHENIX Dock in Map (42). The model was manually adjusted, first by rigid body fitting of domains/regions and subsequently residue-by-residue. Fragments of TSSC4 were manually modeled into unoccupied regions of the cryoEM reconstruction. The model was refined by iterative rounds of real space refinement in PHENIX and manual adjustment in Coot. Manual adjustments also took advantage of the locally refined cryoEM reconstruction. The structural model was evaluated with Molprobity (43). Structure figures were prepared using PyMOL (Version 1.8 Schrödinger, LLC).

### Culturing of HEK293 cells and transient transfection

HEK293 cells were grown in DMEM supplemented with 10% (v/v) fetal bovine serum and 1% (w/v) penicillin/streptomycin (Invitrogen). Transient transfection was performed using Rotifect (Carl Roth) according to the manufacturer's instructions. Briefly, 4.5 × 10^5^ cells were seeded on 6-well plates 24 h prior to transfection. For each well to be transfected, 2 μg plasmid and 5 μl Rotifect were mixed with 250 μl Optimum, and incubated for 5 min at room temperature. Reactions were mixed, incubated for 20 additional minutes at room temperature and then added to the cells. Cells were harvested 48 h after transfection.

### Preparation of nuclear extract

HEK293 cells grown in T75 flasks were harvested in cold PBS and pelleted by centrifugation (1000 × g, 1 min). Cell pellets were re-suspended in 600 μl cold CTX buffer (10 mM HEPES–NaOH, pH 7.9, 1.5 mM MgCl_2_, 10 mM KCl) supplemented with proteinase inhibitors and incubated for 5 min on ice. 600 μl CTX supplemented with 0.2% (v/v) NP-40 were added and, after gentile mixing, the reactions were incubated for another 5 min on ice. Nuclei were pelleted by centrifugation at 4000 × g in a tabletop centrifuge for 3 min. The supernatant (cytosolic fraction) was discarded. Nuclei were re-suspended in 240 μl NX buffer (20 mM HEPES–NaOH, pH 7.9, 1.5 mM MgCl_2_, 420 mM KCl, 0.2 mM EDTA, 25% [v/v] glycerol) supplemented with proteinase inhibitors, and subsequently three times frozen (- 80°C) and thawed (37°C), followed by 1 min of vortexing. After a final centrifugation step at maximum speed for 20 min at 4°C, the supernatant (nuclear extract) was stored at –20°C until further use. The protein concentration was determined *via* Bradford assay.

### Flag-IP and western blot

For Flag-IP and Western blot, 100 μg nuclear extract were incubated with 400 μl RIPA lysis buffer, including 100 mM NaCl, 2% (w/v) BSA and proteinase inhibitors, for 1 h at 4°C on a rotating wheel. Subsequently, 15 μl Flag M2 affinity gel (Sigma Aldrich), equilibrated in RIPA buffer, were added to the reactions and incubated overnight at 4°C on a rotating wheel. The resin was washed four times with RIPA buffer (without BSA) with centrifugation at 4000 × g for 1 min, then re-suspended in 40 μl 2-fold concentrated SDS loading buffer. Samples were heated to 96°C for 5 min, centrifuged at maximum speed for 2 min and the supernatant was then analyzed by SDS-PAGE and western blot using monoclonal anti-Flag M2 antibody (Sigma Aldrich) or rabbit serum containing a polyclonal anti-human SNRNP200 antibody (kind gift by Reinhard Lührmann, Max Planck Institute for Biophysical Chemistry, Göttingen, Germany).

### Generating stable cell lines

Stable Flp-In™ T-REx™ 293 cells for expression of wild type (wt) TSSC4 (TSSC4^wt^) or TSSC4 variant 3 (TSSC4^V3^) with C-terminal 3xFlag-His_6_-Biotin-His_6_ (3xFlag-HBH) tags were generated as described before (44). Transfection of the cell lines was done using Lipofectamine 2000 (Thermo Fisher Scientific). After hygromycin selection, expression of the tagged proteins was confirmed by Western blot using monoclonal anti-Flag M2 antibody.

### Flag-immunoprecipitation followed by mass spectrometry

For mass spectrometric analysis of TSSC4 interactors, Flp-In™ T-REx™ 293 cells stably expressing C-terminally 3xFlag-HBH-tagged TSSC4^wt^ or TSSC4^V3^ (TSSC4^wt^-Flag; TSSC4^V3^-Flag) were grown in T75 flasks, and nuclear extract was prepared as described above. Unmodified Flp-In™ T-REx™ 293 cells were used as a control. 500 μg nuclear extract were incubated with 800 μl immunoprecipitation (IP) buffer (10 mM HEPES–NaOH, pH 7.3, 150 mM NaCl,10 mM MgCl_2_, 10 mM KCl, 0.5 mM EGTA) supplemented with 3 U/ml benzonase and proteinase inhibitors for 1 h on a rotating wheel. For each IP, 50 μl Flag M2 affinity gel, equilibrated in IP buffer, were added to the reaction and incubated overnight at 4°C on a rotating wheel. The resin was washed four times with IP buffer (without supplements) with centrifugation at 4000 × g for 1 min. Bound proteins were eluted by incubation with 50 μl 3xFlag Peptide (Sigma Aldrich) at a concentration of 0.5 μg/μl in 50 mM Tris–HCl, pH 7.5, 150 mM NaCl for 30 min on ice. The supernatant was run on an SDS-PAGE gel until entrance into the separating gel. Coomassie-stained bands were excised and digested with trypsin using a standard protocol (37). After in-gel digestion, peptides were extracted and dried for LC–MS analysis.

Peptides were reconstituted in 15 μl of 0.05% (v/v) TFA, 2% (v/v) acetonitrile and 7 μl were applied to an Ultimate 3000 reversed-phase capillary nano liquid chromatography system connected to a Q Exactive HF mass spectrometer (Thermo Fisher Scientific). Samples were injected and concentrated on a PepMap100 C18 trap column (3 μm, 100 Å, 75 μm i.d. × 2 cm; Thermo Fisher Scientific) equilibrated with 0.05% (v/v) TFA. After switching the trap column inline, LC separations were performed on an Acclaim PepMap100 C18 capillary column (2 μm, 100 Å, 75 μm i.d. × 25 cm; Thermo Fisher Scientific) at an eluent flow rate of 300 nl/min. Mobile phase A contained 0.1% (v/v) formic acid, mobile phase B contained 0.1% (v/v) formic acid in 80% (v/v) acetonitrile. The column was pre-equilibrated with 5% mobile phase B followed by an increase to 44% mobile phase B over 100 min. Mass spectra were acquired in a data-dependent mode, utilizing a single MS survey scan (*m*/*z* 350–1650) with a resolution of 60,000 and MS/MS scans of the 15 most intense precursor ions with a resolution of 15,000. The dynamic exclusion time was set to 20 s and the automatic gain control was set to 3 × 10^6^ and 1 × 10^5^ for MS and MS/MS scans, respectively.

MS and MS/MS raw data were analyzed using the MaxQuant software package (version 2.0.2.0) with implemented Andromeda peptide search engine (45). Data were searched against the human reference proteome downloaded from Uniprot (78,120 proteins; taxonomy 9606; last modified March 7, 2021) using the default parameters and enabling the options label-free quantification (LFQ) and match between runs. Filtering and statistical analysis was carried out using the Perseus software (46). Only proteins that were identified with LFQ intensity values in all 3 replicates within at least one of the three experimental groups were used for downstream analysis. Missing values were replaced from normal distribution (imputation), using the default settings (width 0.3, down shift 1.8). Mean log_2_-fold differences between TSSC4^wt^-Flag-IP or TSSC4^V3^-Flag-IP against control IP were calculated in Perseus using Student's t-tests with false discovery rate-adjusted *P*-values of 0.05.

### FLASH

FLASH was carried out as described before (44). Briefly, Flp-In™ T-REx™ 293 cells expressing either 3xFlag-HBH-tagged TSSC4 or 3xFlag-HBH-tagged GFP were induced with 0.1 μg/ml doxycycline for 16 h and UV-crosslinked with 0.2 mJ/cm^2^ UV-C light. Target proteins were purified using MyONE C1 streptavidin beads (Thermo Fisher Scientific). After a partial RNase digestion using RNase I and end-repair with T4 PNK, uniquely barcoded s-oligos were ligated to each sample (two biological replicates). After several stringent washes (up to 1% [w/v] SDS), biological replicates were merged, bead-bound RNA was released with proteinase K and purified with Oligo Clean and concentrator columns (Zymo Research). RNA was reverse-transcribed and treated with RNase H to phosphorylate 5’-ends of cDNA. The cDNA was then circularized with CircLigaseII (Lucigen), amplified with Q5 polymerase and sequenced on an Illumina platform (100 bp, PE sequencing).

The resulting fastq files were merged with bbmerge, replicates were split using flexbar and mapped to the hg38 assembly of the human genome using bowtie2 and bbmap, after which umi-tools was used to remove PCR duplicates. Enrichment plots were generated using snakePipes (v. 2.5.0) noncoding-RNA-seq workflow, which uses TEtools to calculate the specific enrichment of non-coding RNAs against a background dataset (GFP in this case).

To detect gaps in the sequencing reads indicative of crosslinking sites, reads from FLASH experiments (TSSC4 and GFP) were mapped to U2 or U5 snRNA with bowtie2. After removal of PCR duplicates with umi-tools, each position in U2 or U5 snRNA was analyzed with findallcoverageatposition.jar from Jvarkit to calculate a gap percentage as the ratio of gaps that fall onto a nucleotide over the coverage at the same nucleotide.

## RESULTS

### TSSC4 stably interacts with SNRNP200 and PRPF8^Jab1^

**Figure 1.**
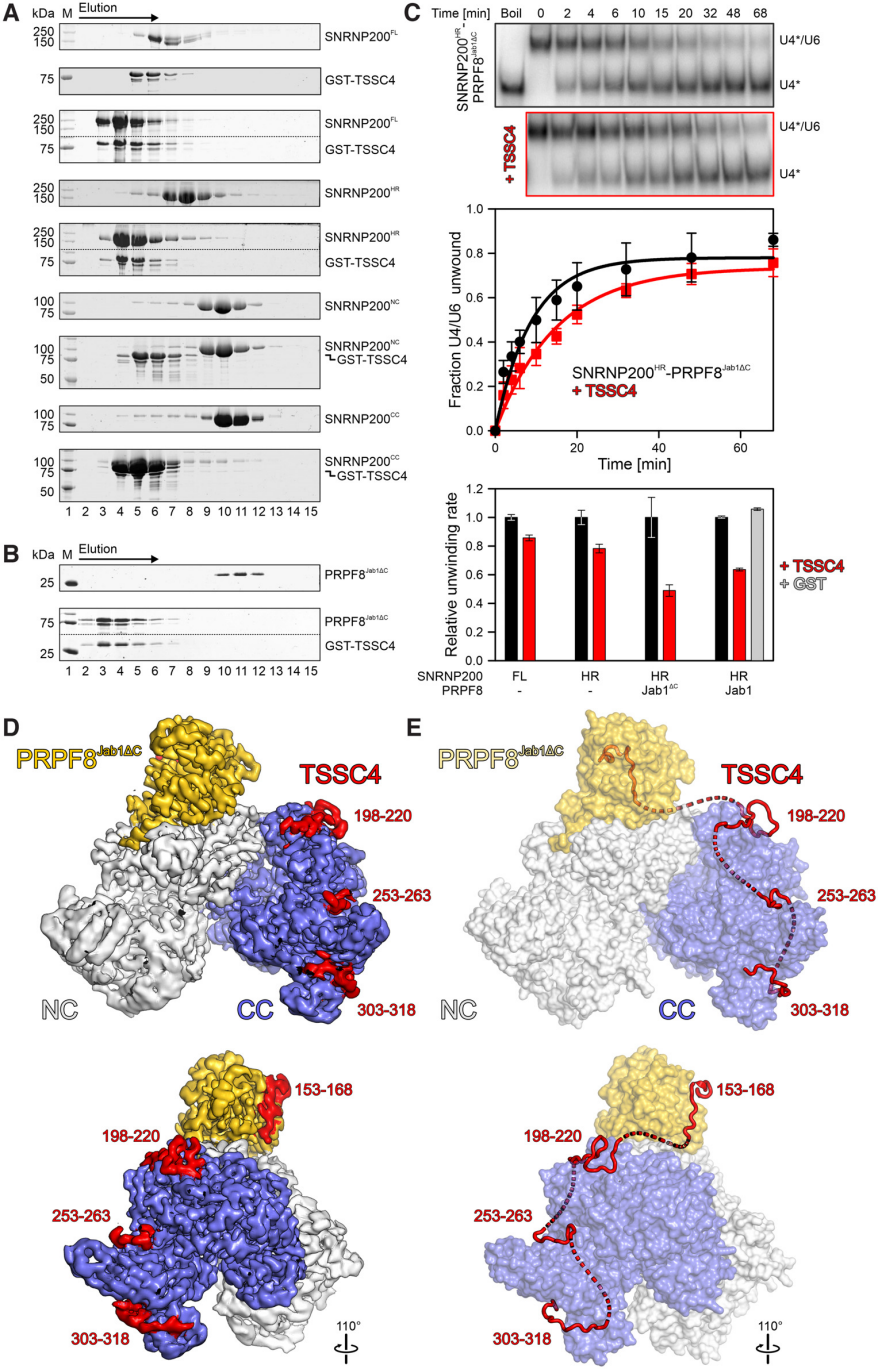
TSSC4 stably interacts with SNRNP200 and PRPF8^Jab1^ and affects SNRNP200 helicase activity. (**A**) SDS-PAGE analysis of elution fractions from analytical SEC, monitoring interaction of GST-TSSC4 with SNRNP200^FL^ and truncation variants (SNRNP200^HR^, SNRNP200^NC^, SNRNP200^CC^). The elution direction is indicated by an arrow above the gels. All runs were conducted under identical conditions and the same elution fractions are shown for each run. Protein bands are identified on the right. M, molecular mass marker. In the third and fifth panel, upper and lower regions of the same gel were spliced together for clarity. Dotted lines, splice positions. (**B**) SDS-PAGE analysis of elution fractions from analytical SEC, monitoring interaction of GST-TSSC4 with PRPF8^Jab1ΔC^, as in (A). (**C**) Top, exemplary non-denaturing PAGE, monitoring SNRNP200^HR^-PRPF8^Jab1^-mediated U4/U6 di-snRNA unwinding in the absence (black outline) or presence (red outline) of TSSC4. Bands are identified on the right. Numbers above the gels, time points after initiation of unwinding at which samples were taken. U4*, radio-labeled U4 snRNA. Middle, quantification of data as shown in the top panel. Data points represent means ± SD; *n* = 3. Data were fit to a single exponential equation (fraction unwound = A [1 – exp(–*k_u_*t)]); A, amplitude of the reaction; *k*_u_, apparent first-order rate constant of unwinding; t, time) using GraphPad Prism. Bottom, relative apparent unwinding rate constants observed for the indicated SNRNP200 variants alone or in complex with PRPF8^Jab1ΔC^ or PRPF8^Jab1^ (as indicated below the bars) and in the absence (black bars) or presence (red bars) of TSSC4. Light gray bar, control with GST instead of TSSC4 added. Data represent means ± SD; *n* = 3. (**D**) Side (top) and back (bottom) views of the cryoEM reconstruction of a SNRNP200^HR^–PRPF8^Jab1ΔC^–TSSC4 complex at 3.05 Å resolution, contoured at 9 root-mean-square deviation (RMSD). SNRNP200 NC, gray; SNRNP200 CC, slate blue; PRPF8^Jab1ΔC^, gold; TSSC4, red. The same coloring is used in the following figures. TSSC4 binding regions are identified by first-last residue numbers. In this and the following figures, rotation symbols represent views relative to (D, top). (**E**) Combined semi-transparent surface (SNRNP200^HR^, PRPF8^Jab1ΔC^) and cartoon (TSSC4) view of the final SNRNP200^HR^–PRPF8^Jab1ΔC^–TSSC4 structural model. Dashed red lines represent residues intervening between SNRNP200^HR^/PRPF8^Jab1ΔC^-binding regions of TSSC4 not resolved in the cryoEM reconstruction.

Co-precipitation analyses have revealed that human TSSC4, a 329-residue protein, exists in complexes containing U5 snRNP proteins, including the spliceosomal SNRNP200 RNA helicase and PRPF8, among others (31). As noted previously (31), fold prediction algorithms (such as FoldIndex; https://fold.proteopedia.org/cgi-bin/findex) suggested that human TSSC4 is largely intrinsically unstructured, and a search with the Basic Logical Alignment Search Tool (https://blast.ncbi.nlm.nih.gov/Blast.cgi) did not reveal any known folded domains. To test if TSSC4 directly binds SNRNP200, we produced recombinant His_6_-GST-TSSC4 (hereafter referred to as GST-TSSC4) in *E. coli* and full-length SNRNP200 (SNRNP200^FL^) *via* recombinant baculoviruses in insect cells, and purified the proteins by native procedures. Tag cleavage led to degradation of the TSSC4 protein, supporting its predicted intrinsic disorder that might render it susceptible to residual proteases, and was therefore omitted for functional analyses *in vitro*. GST-TSSC4 and SNRNP200^FL^ together eluted earlier from an analytical size exclusion chromatography (SEC) column compared to the individual proteins (Figure 1A; Supplementary Figure S1A), indicating the formation of a stable complex. Flag-immunoprecipitation (Flag-IP) *via* a C-terminally Flag-tagged TSSC4 variant (TSSC4^wt^-Flag) produced in transiently transfected HEK293 cells followed by Western blot revealed co-precipitation of SNRNP200 (Supplementary Figure S1B), suggesting that the interaction also ensues *in vivo*.

To further delineate interacting regions of the proteins, we first performed analytical SEC with GST-TSSC4 and different truncation variants of SNRNP200. SNRNP200 comprises an auto-regulatory N-terminal region (residues 1–394) (47), followed by a helicase region (residues 395–2129; SNRNP200^HR^) composed of tandem helicase cassettes (N-/C-terminal cassette, NC/CC) (32). Both helicase cassettes comprise dual RecA-like NTPase domains, a winged helix (WH) domain, a helical bundle (HB) domain, a helix-loop-helix (HLH) domain and an immunoglobulin-like (IG) domain. The latter three domains constitute a Sec63 homology module (48,49). Only the N-terminal cassette (residues 395–1324; SNRNP200^NC^) is an active ATPase/RNA helicase, while the C-terminal cassette (residues 1282–2136; SNRNP200^CC^) is catalytically inactive and serves as an intra-molecular regulator of the NC helicase activity (32,41,50). GST-TSSC4 stably interacted with SNRNP200^HR^ as well as with the isolated CC of SNRNP200, while no stable interaction was observed between GST-TSSC4 and the isolated NC of SNRNP200 (Figure 1A), suggesting that TSSC4 predominantly latches onto the SNRNP200 CC. Next, we performed limited proteolysis of a pre-formed SNRNP200^HR^-GST-TSSC4 complex, followed by analytical SEC. Mass spectrometric analysis revealed that peptides in the C-terminal half of TSSC4 (between residues 149 and 316) co-eluted with SNRNP200^HR^ (Supplementary Figure S1C), which in turn was largely unaffected by the protease treatment as observed before (32). Probing of peptide SPOT arrays, displaying overlapping 25-residue peptides of TSSC4, with SNRNP200^FL^ and truncation variants confirmed an affinity of SNRNP200 variants containing the CC to peptide regions within the C-terminal half of the TSSC4 protein (Supplementary Figure S1D).

Throughout the splicing cycle, SNRNP200 is stably bound to the C-terminal Jab1/MPN domain of PRPF8 (residues 2064–2335; PRPF8^Jab1^) (51–57). PRPF8^Jab1^ can insert a 16-residue C-terminal tail into the SNRNP200 RNA-binding tunnel, thereby inhibiting its helicase activity (33,58). Upon removal of the tail, PRPF8^Jab1^ is converted into a strong activator of the helicase, an effect that can be emulated *via* a PRPF8^Jab1^ variant lacking the C-terminal 16 residues (residues 2064–2319; PRPF8^Jab1ΔC^) (33,58). We, therefore, tested whether TSSC4, in addition to its interaction with the SNRNP200 CC, binds the PRPF8 Jab1 domain. GST-TSSC4 co-migrated with PRPF8^Jab1ΔC^ in analytical SEC, eluting earlier from the column than the individual proteins (Figure 1B), indicating that TSSC4 can stably bind PRPF8^Jab1ΔC^ independent of SNRNP200.

### TSSC4 downregulates SNRNP200 helicase activity

To test if TSSC4 can affect the SNRNP200 RNA helicase activity, we performed radioactive, gel-based U4/U6 di-snRNA unwinding assays with SNRNP200^FL^ or SNRNP200^HR^ in the absence or presence of PRPF8^Jab1^ (SNRNP200 inhibitor), PRPF8^Jab1ΔC^ (SNRNP200 activator) and/or TSSC4. In all tested constellations, TSSC4 exerted an inhibitory effect on SNRNP200-mediated U4/U6 unwinding, which was particularly strong in presence of PRPF8^Jab1^ or PRPF8^Jab1ΔC^, with an up to 50% reduction in the unwinding rate for SNRNP200^HR^-PRPF8^Jab1ΔC^ (Figure 1C). The larger effect of TSSC4 in the presence of PRPF8 Jab1 domain constructs is consistent with its SNRNP200-independent interaction with this PRPF8 region. These results suggest that upon interaction, TSSC4 downregulates SNRNP200 helicase activity, and that by contacting both SNRNP200 and the PRPF8 Jab1 domain, TSSC4 can augment PRPF8^Jab1^-dependent SNRNP200 inhibition and counteract PRPF8^Jab1^-dependent SNRNP200 activation. During U5 snRNP or U4/U6-U5 tri-snRNP assembly and recycling, these TSSC4 activities may help to prevent SNRNP200 from interacting with and unwinding or translocating non-cognate RNAs, or to avert pre-mature SNRNP200-mediated U4/U6 di-snRNA unwinding.

### CryoEM structure of an SNRNP200^HR^-PRPF8^Jab1^^Δ^^C^-TSSC4 complex

**Figure 2.**
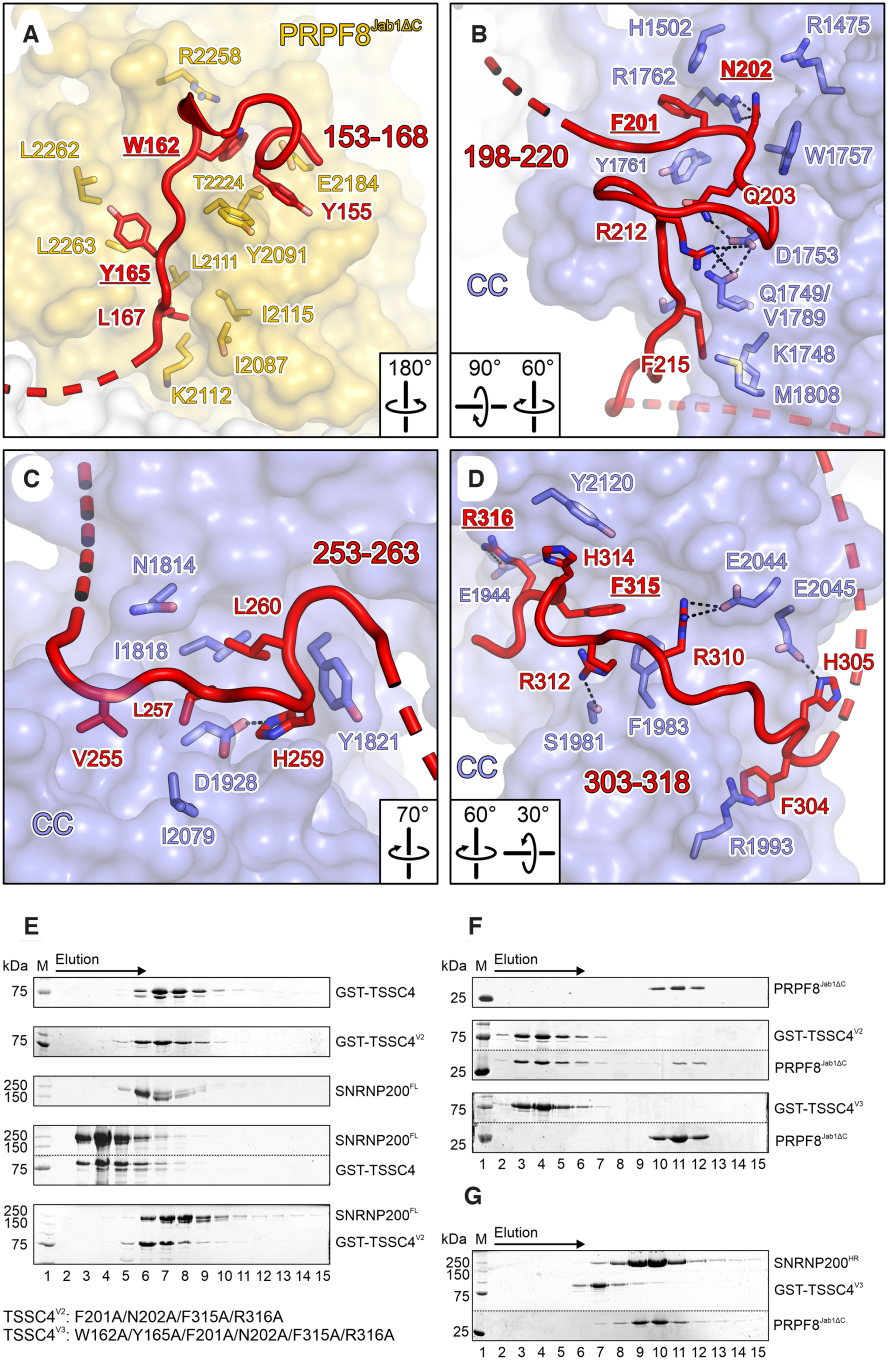
Detailed TSSC4-PRPF8^Jab1^/SNRNP200^HR^ interactions and design of a non-binding TSSC4 variant. (**A–D**) Close-up views of the TSSC4 binding regions in the SNRNP200^HR^-PRPF8^Jab1ΔC^-TSSC4 structure. PRPF8^Jab1ΔC^ and SNRNP200^HR^ are shown as semi-transparent surfaces. TSSC4 regions are shown as cartoon. Selected interface residues are shown a sticks colored by atom type and labeled. In this and the following figures: Carbon, as the respective protein; nitrogen, blue; oxygen, red; sulfur, yellow; dashed black lines, hydrogen bonds or salt bridges. Labels of TSSC4 residues that were altered to alanines in TSSC4 variants 1, 2 or 3 are in bold and underlined. (**E**) SDS-PAGE analysis of elution fractions from analytical SEC, monitoring interaction of GST-TSSC4^V2^ (F201A/N202A/F315A/R316A) with SNRNP200^FL^, compared to GST-TSSC4^wt^. All runs were conducted under identical conditions and the same elution fractions are shown for each run. Protein bands are identified on the right. M, molecular mass marker. In the fourth panel, upper and lower regions of the same gel were spliced together for clarity. Dotted lines, splice positions. (**F**) SDS-PAGE analysis of elution fractions from analytical SEC, monitoring the interaction of GST-TSSC4^V2^ and GST-TSSC4^V3^ (W162A/Y165A/F201A/N202A/F315A/R316A) with PRPF8^Jab1ΔC^, as in (E). In second and third panel, upper and lower regions of the same gel were spliced together for clarity. Dotted lines, splice positions. (**G**) SDS-PAGE analysis of elution fractions from analytical SEC, monitoring the interaction of GST-TSSC4^V3^ with a SNRNP200^HR^-PRPF8^Jab1ΔC^ complex, as in (E).

Co-purification of SNRNP200^HR^ and GST-TSSC4 enabled tag cleavage without degradation of the TSSC4 protein. Subsequent addition of PRPF8^Jab1ΔC^ yielded a ternary SNRNP200^HR^–PRPF8^Jab1ΔC^–TSSC4 complex. We determined a cryoEM structure of this ternary complex at a nominal resolution of 3.05 Å (Supplementary Table S1; Supplementary Figure S2; Supplementary Figure S3). As in previously published crystal structures of SNRNP200^HR^ in complex with PRPF8^Jab1^ (33) or PRPF8^Jab1ΔC^ (41), the Jab1^ΔC^ domain resides on top of the SNRNP200 NC (Figure 1D, E). The cryoEM map unequivocally revealed four short regions in the C-terminal half of TSSC4 that contact PRPF8^Jab1ΔC^ or SNRNP200^HR^, consistent with our interaction assays. TSSC4 region 1 (residues 153–168) lies along one flank of PRPF8^Jab1ΔC^, with aromatic and hydrophobic residues of TSSC4 (Y155, W162, Y165, L167) engaging in stacking, cation-π and hydrophobic interactions with PRPF8 residues (Figure 2A). TSSC4 portions C-terminal of region 1 meander along the backside of the SNRNP200 CC. Region 2 (residues 198–220) interacts predominantly with the WH domain of the SNRNP200 CC *via* a mixture of aromatic stacking (F201 to SNRNP200 H1502, Y1761), hydrogen bonding/salt bridge (N202, Q203, R212 to SNRNP200 R1762, D1753, D1753/Q1749, respectively) and hydrophobic interactions (F215 to SNRNP200 K1748, V1789, M1808; Figure 2B). Region 3 (residues 253–263) comes to rest in a groove formed between the WH, HB and IG domains of the SNRNP200 CC, where it engages in hydrophobic (V255, L257, L260 to SNRNP200 I2079, I1818/I2079, I1818/Y1821, respectively) and hydrogen bond contacts (H259 to SNRNP200 D1928; Figure 2C). Finally, region 4 (residues 303–318) occupies a shallow groove between the HLH and IG domains of the CC Sec63 module, forming cation-π interactions (F304, R312 to SNRNP200 R1993, F1983, respectively), salt bridges (H305, R310, R316 to SNRNP200 E2045, E2044, E1944, respectively), hydrogen bonds (R312 to SNRNP200 S1981) and aromatic stacking interactions (H314, F315 to SNRNP200 Y2021, F1983, respectively; Figure 2D). Although the TSSC4 interaction regions are short, being disordered they present most of their molecular surfaces to the environment and interact with PRPF8^Jab1ΔC^ or SNRNP200^HR^*via* considerable interface areas (PRPF8^Jab1ΔC^–TSSC4 region 1, 725 Å^2^; SNRNP200^HR^-TSSC4 region 2, 873 Å^2^; SNRNP200^HR^-TSSC4 region 3, 526 Å^2^; SNRNP200^HR^-TSSC4 region 4, 895 Å^2^; according to the PISA server; https://www.ebi.ac.uk/pdbe/pisa) (59). A multiple sequence alignment of TSSC4 homologs revealed that the PRPF8^Jab1ΔC^-binding region 1 and SNRNP200^HR^-binding regions 2 and 4 of TSSC4 are highly conserved, with a lower level of conservation in SNRNP200^HR^-binding region 3 (Supplementary Figure S4), suggesting that the observed mode of interaction also ensues in other organisms that harbor a TSSC4 ortholog.

Consistent with the predicted intrinsic disorder of TSSC4, all bound regions of TSSC4, while immobilized, maintain irregular structures, revealing no induced secondary structure elements upon SNRNP200^HR^-PRPF8^Jab1ΔC^ binding. TSSC4 regions outside of the four SNRNP200^HR^/PRPF8^Jab1ΔC^-binding regions, although contained in the complex, could not be located in the cryoEM reconstruction, suggesting that they loop out before (residues 1–152), between (residues 169–197, 221–252, 264–302; red dashed lines in Figure 1E) or behind (residues 319–329) the bound regions and remain disordered. The distal edges of the region 1-binding site on PRPF8^Jab1ΔC^ and the region 4-binding site on SNRNP200^HR^ are >120 Å apart from each other, revealing that its intrinsic disorder allows TSSC4 to bridge large distances on the SNRNP200^HR^-PRPF8^Jab1ΔC^ complex *via* its C-terminal half alone. The independent, stable interaction of TSSC4 with SNRNP200^HR^ and PRPF8^Jab1^ and the binding of these proteins *via* several short regions suggest that TSSC4 may kinetically aid SNRNP200-PRPF8 complex formation, by increasing the local concentration of one interaction partner in the vicinity of the other. TSSC4 may also help to spatially arrange the proteins during U5 snRNP assembly, by restricting the extent to which the two proteins can spatially separate.

### Design of a TSSC4 variant deficient in SNRNP200^HR^–PRPF8^Jab1^ binding

To probe the importance of interacting regions and to develop a tool for further studying TSSC4 functions, we attempted to design a TSSC4 variant that is deficient in SNRNP200 and PRPF8^Jab1^ binding. To disrupt the TSSC4-PRPF8^Jab1^ interaction, we jointly exchanged TSSC4 W162 and Y165 to alanines (TSSC4 variant 1; TSSC4^V1^). W162 and Y165 of TSSC4 engage in cation-π and hydrophobic contacts with PRPF8^Jab1^ residues (Figure 2A) and are highly conserved among TSSC4 orthologs (Supplementary Figure S4). To abolish the TSSC4–SNRNP200^HR^ interaction, we focused on TSSC4 binding regions 2 (residues 198–220) and 4 (residues 303–318), which are more conserved among TSSC4 orthologs compared to binding region 3 (Supplementary Figure S4). We jointly exchanged F201 and N202 (region 2; aromatic stacking, cation–π and hydrogen bonded interactions) as well as F315 and R316 (region 4; aromatic stacking, cation-π and salt bridge interactions) for alanines, generating TSSC4 variant 2 (TSSC4^V2^; Figures 2B, D). We produced and purified TSSC4^V2^ as well as a TSSC4 variant 3 that combines the residue exchanges of TSSC4^V1^ and TSSC4^V2^. As revealed by analytical SEC, TSSC4^V2^ did not interact with SNRNP200^FL^ (Figure 2E) but retained interaction with PRPF8^Jab1ΔC^ (Figure 2F). TSSC4^V3^ bound neither of the individual proteins nor the SNRNP200^HR^–PRPF8^Jab1ΔC^ complex (Figure 2F, G).

### Structural comparisons suggest a role of TSSC4 in remodeling AAR2-bound assembly intermediates

**Figure 3.**
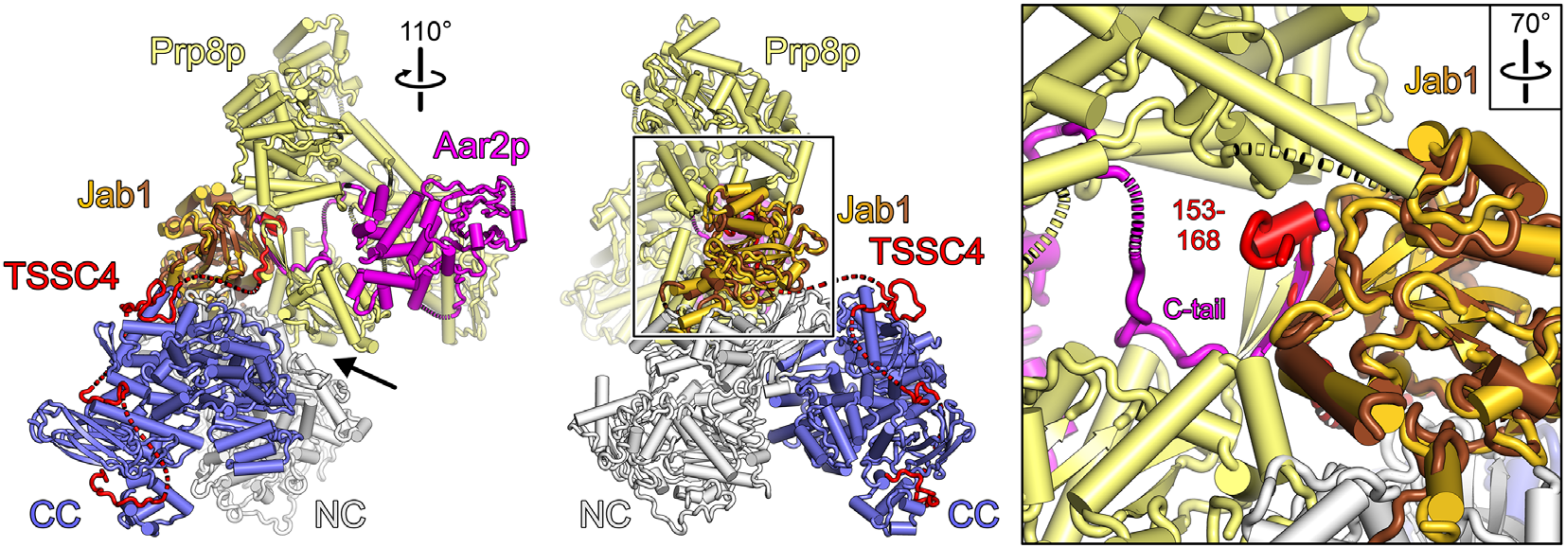
Structural comparison of the SNRNP200^HR^–PRPF8^Jab1ΔC^–TSSC4 complex complex to a Prp8p–Aar2p complex. Superposition of a crystal structure of the yeast Prp8p–Aar2p complex (PDB ID 4I4) (60) on the SNRNP200^HR^–PRPF8^Jab1ΔC^–TSSC4 complex according to the Prp8p/PRPF8 Jab1 domains. Prp8p excluding the Jab1 domain, pale yellow; Prp8p Jab1 domain, brown; Aar2p, magenta. Left and middle, overview in two orientations (SNRNP200^HR^–PRPF8^Jab1ΔC^–TSSC4 oriented as in Figure 1D). The rotated view (left) reveals steric conflicts between SNRNP200 and regions of Prp8p (black arrow). Right, close-up view on the C-terminal tail (C-tail) of Aar2p and TSSC4 region 1 (residues 153–168) bound in steric conflict at equivalent sites of the respective Prp8p/PRPF8 Jab1 domain (parts of Prp8p cut away).

Previously, a crystal structure of yeast Prp8p in complex with the U5 snRNP assembly factor Aar2p has been presented (60). The human Aar2p ortholog, AAR2, has been shown to also interact with PRPF8 domains (27), suggesting that it might bind in a similar manner to PRPF8 as observed for the yeast orthologs. Furthermore, AAR2 and TSSC4 have been found to co-exist in complexes containing U5-specific proteins (26,28,31). We, therefore, compared our SNRNP200^HR^–PRPF8^Jab1ΔC^–TSSC4 cryoEM structure to the Prp8p–Aar2p complex structure (PDB ID 4I43) (60) by superimposing *via* the Prp8/PRPF8 Jab1 domains (Figure 3). The comparison revealed that the presumed position of the PRPF8 Jab1 domain in a yeast-like PRPF8-AAR2 complex is incompatible with concomitant binding of this domain to SNRNP200 and TSSC4 (Figure 3). Moreover, the comparison suggested that the AAR2 C-terminal tail may occupy the same binding site on the Jab1 domain as TSSC4 region 1 (Figure 3, close-up). We, therefore, suggest that TSSC4 induces remodeling of an initially formed PRPF8–AAR2 complex (or PRPF8–EFTUD2–SNRNP40–AAR2 complex; modeling suggested that binding sites on PRPF8 for EFTUD2 and SNRNP40 remain accessible in presence of AAR2), leading to the formation of a PRPF8–SNRNP200–AAR2–TSSC4 (or PRPF8–SNRNP200–EFTUD2–SNRNP40–AAR2–TSSC4) complex, in which the tetrameric U5 sub-module or parts thereof are productively assembled.

### Binding of the SNRNP200 CC by FBP21, C9ORF78 and TSSC4 is mutually exclusive

**Figure 4.**
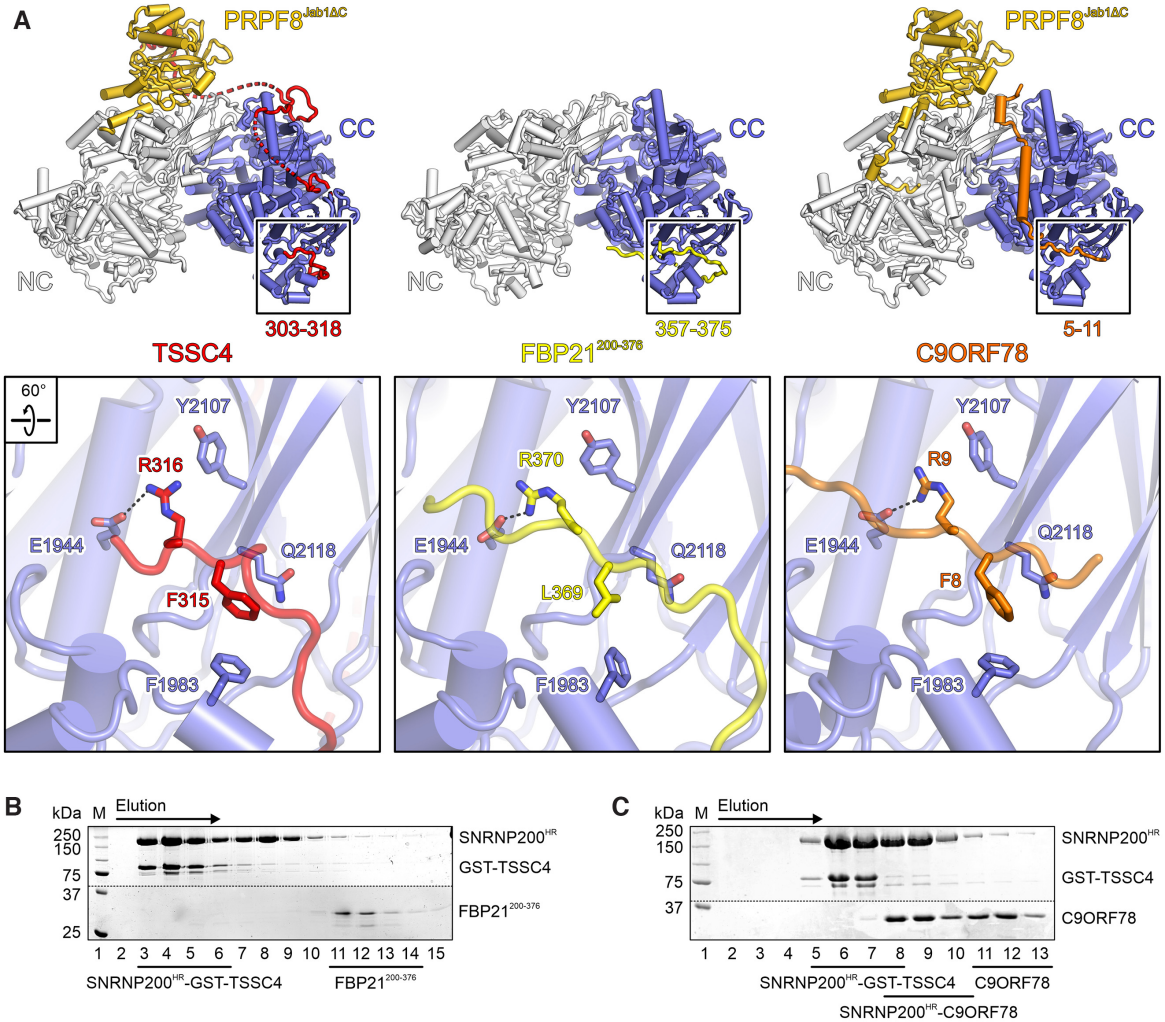
Mutually exclusive binding of TSSC4, FBP21 and C9ORF78 to SNRNP200. (**A**) Top, side-by-side comparison of SNRNP200^HR^-PRPF8^Jab1ΔC^-TSSC4 (left, this work), SNRNP200^HR^–FBP21^200–376^ (middle; PDB ID 7OS1) and SNRNP200^HR^–PRPF8^Jab1^–C9ORF78 (right; PDB ID 7OS2) (35) complexes. FBP21^200–376^, yellow; C9ORF78, orange. Bottom, close-up views of the common binding sites of the three IDPs at the C-terminal Sec63 module of SNRNP200^HR^ (boxed in the top images). Selected interacting residues are shown as sticks colored by atom type and labeled. (B, C) SDS-PAGE analysis of elution fractions from analytical SEC of SNRNP200^HR^/FBP21^200–376^/TSSC4 (**B**) SNRNP200^HR^/C9ORF78/GST-TSSC4 (**C**) mixtures. Different elution fractions were analyzed in (B) and (C). Elution fractions containing complexes or isolated proteins are identified at the bottom. The elution direction is indicated by an arrow above the gels. Protein bands are identified on the right. M, molecular mass marker. Different regions of the same gels were spliced together for clarity. Dotted lines, splice positions.

We have previously characterized the interaction of SNRNP200 with two splicing factors that are also largely intrinsically disordered, FBP21 and C9ORF78 (34,35). FBP21 enters the spliceosome during formation of the pre-catalytic B complex together with seven additional B-specific proteins and leaves the spliceosome again during the following activation step (61), likely facilitating ordered transitions toward a catalytically competent spliceosome. C9ORF78 is recruited to the spliceosome at a later stage and has been found associated with spliceosome preparations enriched for the step 1 spliceosome (C complex) (61), regulating usage of alternative 3’-splice sites and other alternative splicing events (35). Like TSSC4, both proteins also contact the SNRNP200 CC (34,35). Notably, the binding site of TSSC4 binding region 4 (residues 303–318) overlaps with a binding site of FBP21 and C9ORF78 regions at the Sec63 unit of the SNRNP200 CC (Figure 4A). Moreover, all three proteins employ an F-R (F315-R316 in TSSC4; F8-R9 in C9ORF78) or L-R (L369-R370 in FBP21) motif to engage in similar contacts with the F1983, E1944 and E2119 residues of SNRNP200^HR^ (Figure 4A). This observation supports the idea that the SNRNP200 CC serves as a latching point for diverse intrinsically disordered proteins (IDPs) that either function during snRNP assembly (TSSC4) or during different stages of the splicing cycle (FBP21, C9ORF78).

Mutually exclusive binding of the three proteins to SNRNP200 may serve to prevent premature interactions during U5 snRNP assembly (TSSC4) or support ordered recruitment of regulatory proteins during splicing (FBP21 and C9ORF78). To test this notion, we monitored binding competition among TSSC4, FBP21 and C9ORF78. Instead of full-length FBP21, we employed a C-terminal fragment (FBP21^200–376^) that encompasses the SNRNP200 CC-binding region (34,35). We mixed pre-formed SNRNP200^HR^–FBP21^200–376^ or SNRNP200^HR^–C9ORF78 complexes with GST-TSSC4 in estimated equimolar ratios and subjected the mixtures to analytical SEC. In neither case a ternary complex ensued and in both cases SNRNP200^HR^ preferentially associated with GST-TSSC4 (Figure 4B, C). FBP21^200–376^ was fully displaced by GST-TSSC4 (Figure 4B) while, due to an excess of SNRNP200^HR^–C9ORF78 over GST–TSSC4 in case of the SNRNP200^HR^–C9ORF78/GST–TSSC4 mixture, a small amount of binary SNRNP200^HR^–C9ORF78 complex eluted from the SEC column in addition to SNRNP200^HR^–GST–TSSC4 and isolated C9ORF78 (Figure 4C). These observations indicate that TSSC4 exhibits a higher affinity to SNRNP200^HR^ compared to FBP21^200–376^ or C9ORF78 and prevents binding of the latter two proteins. Thus, during U5 snRNP assembly, TSSC4 appears to occupy a functionally important site on SNRNP200 to prevent premature association of the helicase with splicing factors that are to be recruited only during specific phases of the splicing cycle.

### Disruption of the TSSC4 interactions with SNRNP200^HR^–PRPF8^Jab1^^Δ^^C^ leads to altered interactions of TSSC4 with splicing factors, snRNP assembly factors and chaperones

**Figure 5.**
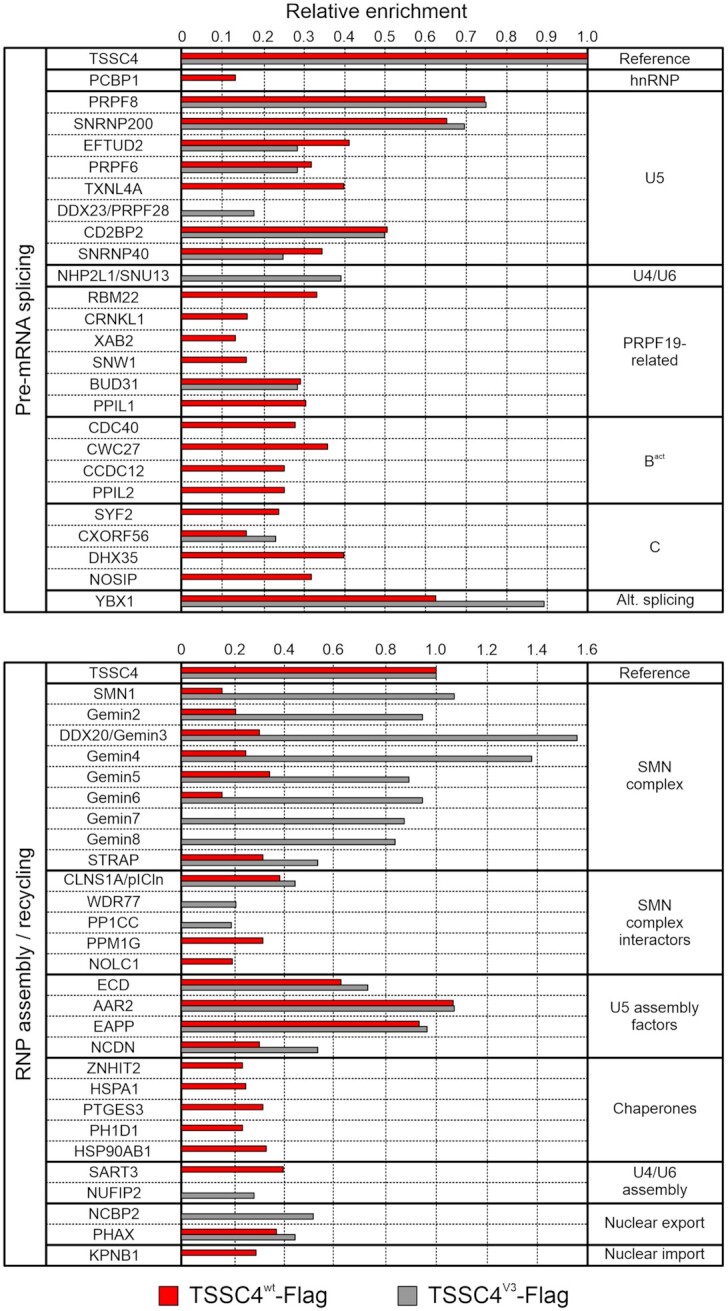
Distinct protein interactomes of TSSC4^wt^ and the non-SNRNP200^HR^/PRPF8^Jab1^-binding variant TSSC4^V3^. Spliceosomal (top) and snRNP assembly/recycling (bottom) proteins significantly enriched (log_2_-fold change ≥ 1 and FDR-adjusted *P*-value ≤ 0.05) in Flag-IPs from nuclear extract of HEK293 cells stably expressing TSSC4^wt^-Flag (red) or TSSC4^V3^-Flag (gray). Data represent mean log_2_-fold changes (*n* = 3) of protein intensities of TSSC4^WT^-Flag-IP or TSSC4^V3^-Flag-IP relative to the control Flag-IP, normalized to the log_2_-fold change of the respective TSSC4 variant. All co-precipitated proteins are listed in Supplementary Table S2.

**Figure 6.**
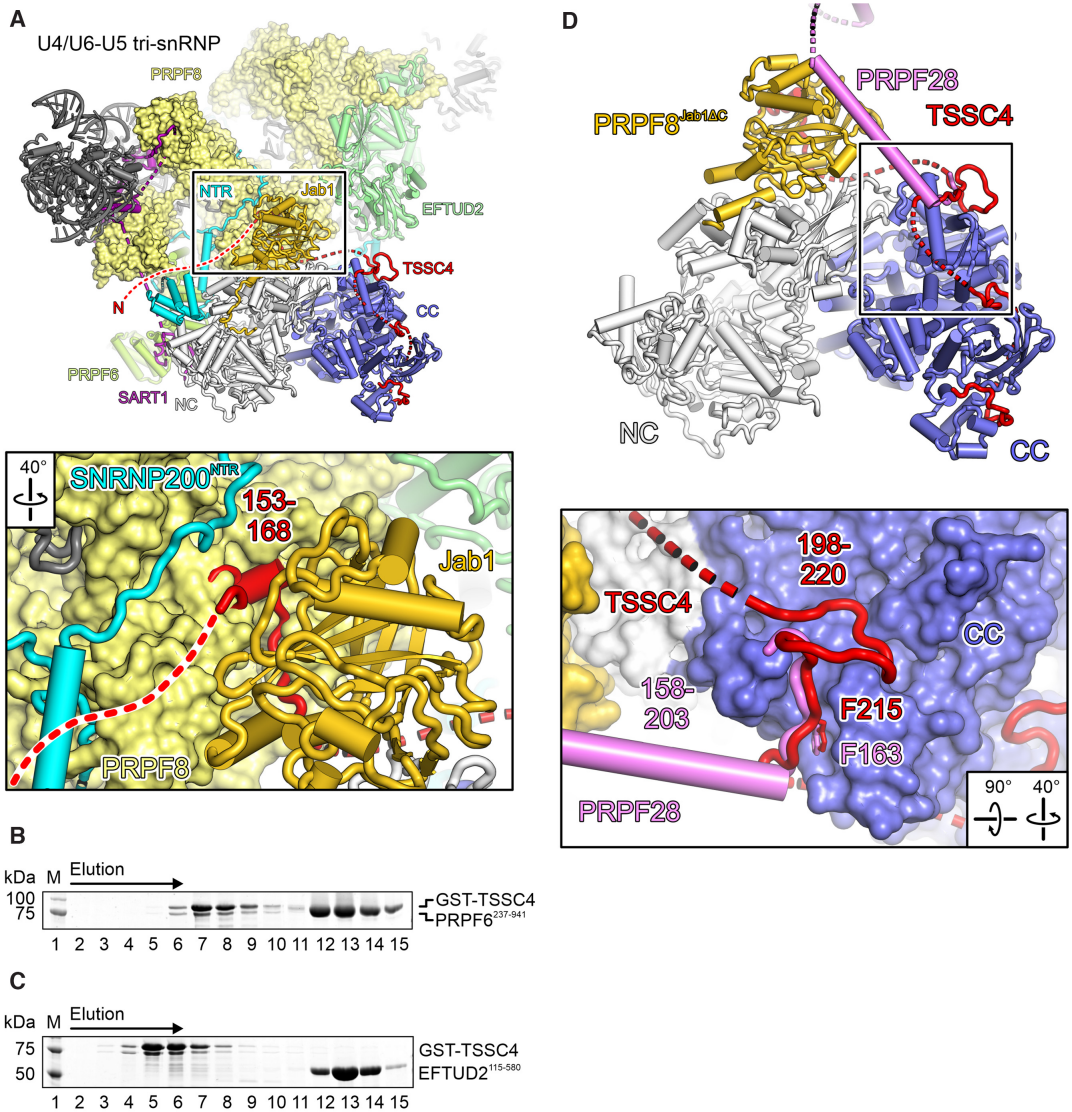
Structural comparison of the SNRNP200^HR^–PRPF8^Jab1ΔC^–TSSC4 complex complex to the U4/U6–U5 tri-snRNP. (**A**) Superposition of the SNRNP200^HR^–PRPF8^Jab1ΔC^–TSSC4 complex structure on the structure of a human U4/U6–U5 tri-snRNP (PDB ID 6QW6) (51) according to the SNRNP200 helicase region. SNRNP200 N-terminal region (SNRNP200^NTR^), cyan; EFTUD2, pale green; PRPF6, lime green; SART1 (Snu66 in yeast), purple; other U4/U6-U5 tri-snRNP, dark gray. The TSSC4 N-terminal region, not resolved in our cryoEM map, is shown as a red dashed line in an arbitrary location. Close-up, in the mature U4/U6-U5 tri-snRNP, PRPF8^Jab1ΔC^-binding region 1 of TSSC4 would intervene between the PRPF8 Jab1 domain and other parts of PRPF8, giving rise to minor sterical conflicts. (**B**,**C**) SDS-PAGE analysis of elution fractions from analytical SEC, monitoring interaction of GST-TSSC4 with PRPF6^237–941^ (**B**) or EFTUD2^115–580^ (**C**). Different elution fractions were analyzed in (B) and (C). Protein bands are identified on the right. M, molecular mass marker. (**D**) Superposition of the SNRNP200^HR^/PRPF8^Jab1^ region in complex with an isolated helix of the PRPF28 protein from a human U4/U6–U5 tri-snRNP structure (PDB ID 6QW6) (51) with the SNRNP200^HR^-PRPF8^Jab1ΔC^–TSSC4 complex structure. PRPF28, violet. Close-up, PRPF28 residues 158–165 occupy the same binding site on SNRNP200^CC^ as the TSSC4 region 2.

For comparative protein interactome studies, we generated HEK293 cell lines stably expressing C-terminally Flag-tagged TSSC4^wt^-Flag or TSSC4^V3^-Flag, and conducted Flag-IPs from nuclear extracts, followed by mass spectrometric identification and label-free quantification of co-purified proteins. Flag-IPs from nuclear extracts of non-transfected cells were used as a control to differentiate specific enrichment from non-specific background (e.g., proteins remaining bound to the beads despite extensive washing). In total, we identified and quantified 2,596 proteins (Supplementary Table S2). Proteins with at least a 2-fold increase in relative intensity compared to the control (log2-fold change ≥ 1) and a FDR-adjusted *P*-value ≤ 0.05 were considered significantly enriched (1,239 proteins for TSSC4^wt^-Flag-IP; 798 proteins for TSSC4^V3^-Flag-IP; 424 proteins common to both IPs). TSSC4^wt^-Flag and TSSC4^V3^-Flag were significantly enriched in both experiments against the control (TSSC4^wt^-Flag log_2_-fold change = 7.5; TSSC4^V3^-Flag log_2_-fold change = 5.7). For comparison of the TSSC4^wt^-Flag and TSSC4^V3^-Flag interactomes, we focused on all known spliceosomal and snRNP assembly/recycling proteins that were significantly enriched in our datasets and calculated their relative enrichment by normalization of their log_2_-fold changes to the log_2_-fold changes of TSSC4^wt^-Flag or TSSC4^V3^-Flag (Figure 5). Although TSSC4^V3^ did not interact with SNRNP200 or PRPF8^Jab1ΔC^*in vitro*, SNRNP200 and PRPF8 were almost equally enriched in both Flag-IPs (Figure 5). As also all other U5 snRNP-specific proteins were co-precipitated to a similar extent *via* TSSC4^wt^-Flag and TSSC4^V3^-Flag (except PRPF28/DDX23, which was only significantly enriched *via* TSSC4^V3^-Flag), SNRNP200 and PRPF8 may have been indirectly captured through their interaction with these U5 factors in the TSSC4^V3^-Flag-IP. Alternatively, or in addition, TSSC4 may exhibit additional binding sites on other regions of PRPF8 and/or SNRNP200, not resolved in our complex structure. Interestingly, both TSSC4^wt^-Flag and TSSC4^V3^-Flag co-precipitated CD2BP2, a U5-specific protein that leaves upon U4/U6–U5 tri-snRNP formation, to a similar extent, confirming a role of TSSC4 in U5 snRNP assembly. The TXNL4 protein that directly binds CD2BP2 but remains upon tri-snRNP formation, on the other hand, was selectively enriched only *via* TSSC4^wt^-Flag, suggesting that TSSC4 may be involved in sorting factors that remain or leave upon association of U5 snRNP with U4/U6 di-snRNP. Conversely, the U4/U6 protein, NHP2L1/SNU13, was selectively enriched only *via* TSSC4^V3^-Flag, possibly indicating that TSSC4 needs to be displaced from its SNRNP200/PRPF8^Jab1^ binding sites upon tri-snRNP formation. Consistent with the latter notion, steric conflicts, albeit minor, were observed upon alignment of our SNRNP200^HR^–PRPF8^Jab1ΔC^-TSSC4 structure with a cryoEM structure of the human U4/U6–U5 tri-snRNP (PDB ID 6QW6; Figure 6A; Supplementary File S1), and TSSC4 has not been found in a cryoEM reconstruction of the tri-snRNP (51).

Assuming that during U5 snRNP or U4/U6–U5 tri-snRNP assembly intermediate(s) ensue that still contain TSSC4 and resemble (parts of) the U4/U6–U5 tri-snRNP, the 152 N-terminal TSSC4 residues not resolved in our structure might interact with domains of PRPF8, the SNRNP200 N-terminal region and/or U5 snRNP proteins EFTUD2 or PRPF6 (Figure 6A). Our *in vitro* interaction assays did not reveal any stable interaction of TSSC4 with the SNRNP200 N-terminal region, and GST-TSSC4 did not stably interact with recombinantly produced PRPF6^237–941^ or EFTUD2^115–580^ (regions in closest proximity of TSSC4 in the above superposition) in analytical SEC (Figure 6B, C). These observations indirectly support the idea that TSSC4 exhibits additional contacts with PRPF8 beyond its interaction with the PRPF8^Jab1^ domain.

In the U4/U6–U5 tri-snRNP (PDB ID 6QW6) and in the pre-B complex spliceosome (PDB ID 6QX9) (51), an isolated α-helix and neighboring loops of the PRPF28 RNA helicase (residues 158–203) bridge the SNRNP200 CC and the PRPF8 Jab1 domain (Figure 6D). A detailed comparison with our SNRNP200^HR^-PRPF8^Jab1ΔC^–TSSC4 complex structure revealed that PRPF28 residues 158–165 occupy part of the binding site on the SNRNP200 CC that also can accommodate TSSC4 region 2 (residues 198–220; Figure 6D, close-up), which has been destroyed in TSSC4^V3^. In both cases, a phenylalanine residue (TSSC4 F215, PRPF28 F163) occupies the same binding pocket on SNRNP200^CC^ (Figure 6D, close-up). The observation that TSSC4^V3^-Flag, but not TSSC4^wt^-Flag, co-precipitated PRPF28 suggests that the binding regions altered in TSSC4^V3^ indeed fail to associate with SNRNP200 or PRPF8^Jab1^ in the cellular assays. Furthermore, this finding extends the notion that one function of TSSC4 during U5 snRNP or U4/U6–U5 tri-snRNP assembly is the prevention of pre-mature interactions with later-interacting proteins, in this case the PRPF28 helicase.

While contrary to a previous analysis (31) we did not detect PRPF19 complex proteins significantly enriched in our IPs, several PRPF19-related proteins co-purified with TSSC4^wt^-Flag (Figure 5). Most of these proteins were not significantly enriched in the TSSC4^V3^-Flag-IP (Figure 5), suggesting that their associations with TSSC4 are indirect and depend on stable binding of TSSC4 to PRPF8 and/or SNRNP200. The PRPF19-related protein, BUD31, was equally enriched in the TSSC4^wt^-Flag-IP and TSSC4^V3^-Flag-IPs, possibly hinting at a direct interaction with TSSC4 independent of SNRNP200 and PRPF8. As in human the U5 snRNP is profoundly remodeled during splicing and emerges after the splicing reaction associated with the PRPF19 complex and additional proteins (17), these observations further corroborate a suggested role of TSSC4 in U5 snRNP recycling after splicing (31).

Several splicing factors that first enter the spliceosome during the B^act^ and C complex stages were significantly enriched selectively in the TSSC4^wt^-Flag-IP (except CXORF56 that was enriched in both IPs; Figure 5), suggesting that TSSC4 interactions with these proteins to a large extent depend on TSSC4-PRPF8/SNRNP200 interactions. This observation again supports a role of TSSC4 in U5 snRNP recycling, which may be initiated by PRPF8/SNRNP200-dependent interaction of TSSC4 with post-catalytic spliceosomes (P complex) that contain most of the co-precipitated B^act^ and C complex proteins.

Consistent with a role of TSSC4 as a U5 snRNP and U4/U6–U5 tri-snRNP assembly/recycling factor, our Flag-IPs enriched many proteins known to be involved in snRNP biogenesis, as well as chaperone machinery previously implicated in snRNP assembly or recycling (Figure 5). Similar observations have been made by others (31). Among the group of snRNP assembly/recycling factors, several subunits of the SMN complex were co-precipitated *via* TSSC4^wt^-Flag (Figure 5). The SMN complex supports the ordered formation of Sm core RNPs during the cytoplasmic phase of *de novo* snRNP biogenesis (8,9,12). It also exhibits nuclear fractions that are localized to Cajal bodies and Gemini bodies (Gems), and plays a role in the formation of these membrane-less compartments (62–65). While the precise functions of nuclear-localized SMN complex are presently not clear, the Cajal body-localized fraction is most likely involved in snRNP recycling after splicing, which has been suggested to involve trafficking of snRNP components through Cajal bodies (14,15). The TSSC4 interaction with SMN complex components may thus be indicative of a role of TSSC4 in trafficking U5 snRNP or U4/U6–U5 tri-snRNP assembly/recycling intermediates, such as the U5 tetrameric sub-module, to Cajal bodies.

Strikingly, the interaction pattern of the non-SNRNP200^HR^/PRPF8^Jab1ΔC^-binding TSSC4^V3^-Flag with different categories of snRNP assembly factors/chaperones is altered in a systematic fashion compared to that of TSSC4^wt^-Flag. Compared to TSSC4^wt^-Flag, TSSC4^V3^-Flag exhibited enhanced interactions with all subunits of the SMN complex, largely unaltered interaction with U5 snRNP assembly factors and abrogation of the interactions with chaperone machinery implicated in snRNP assembly (Figure 5). The differential effects of disturbed SNRNP200/PRPF8 interactions of TSSC4 on different fractions of the molecular snRNP biogenesis machinery may reflect different stages of U5 snRNP and U4/U6–U5 tri-snRNP assembly or recycling involving (i) TSSC4 and the SMN complex, (ii) TSSC4 and U5 assembly factors and (iii) TSSC4 and assembly chaperones.

### TSSC4 UV-crosslinks with U2 and U5 snRNAs

Apart from the requirement to organize numerous protein–protein interactions, proteins or protein complexes have to be accurately placed on snRNAs during snRNP assembly and recycling. To test whether TSSC4 might also be involved in organizing protein-RNA interactions during these processes, we addressed the question if TSSC4 directly interacts with RNAs by performing UV-crosslinking followed by enrichment of TSSC4-bound RNAs and their identification *via* RNA sequencing (RNAseq). To this end, we employed our HEK293 cell line stably expressing Flag-tagged TSSC4^wt^ and Fast Ligation of RNA after some sort of Affinity Purification for High-throughput Sequencing (FLASH) (44). Green fluorescent protein (GFP)-expressing cells served as a negative control. RNAseq yielded 3.2M reads after filtering PCR duplicates and low quality reads.

**Figure 7.**
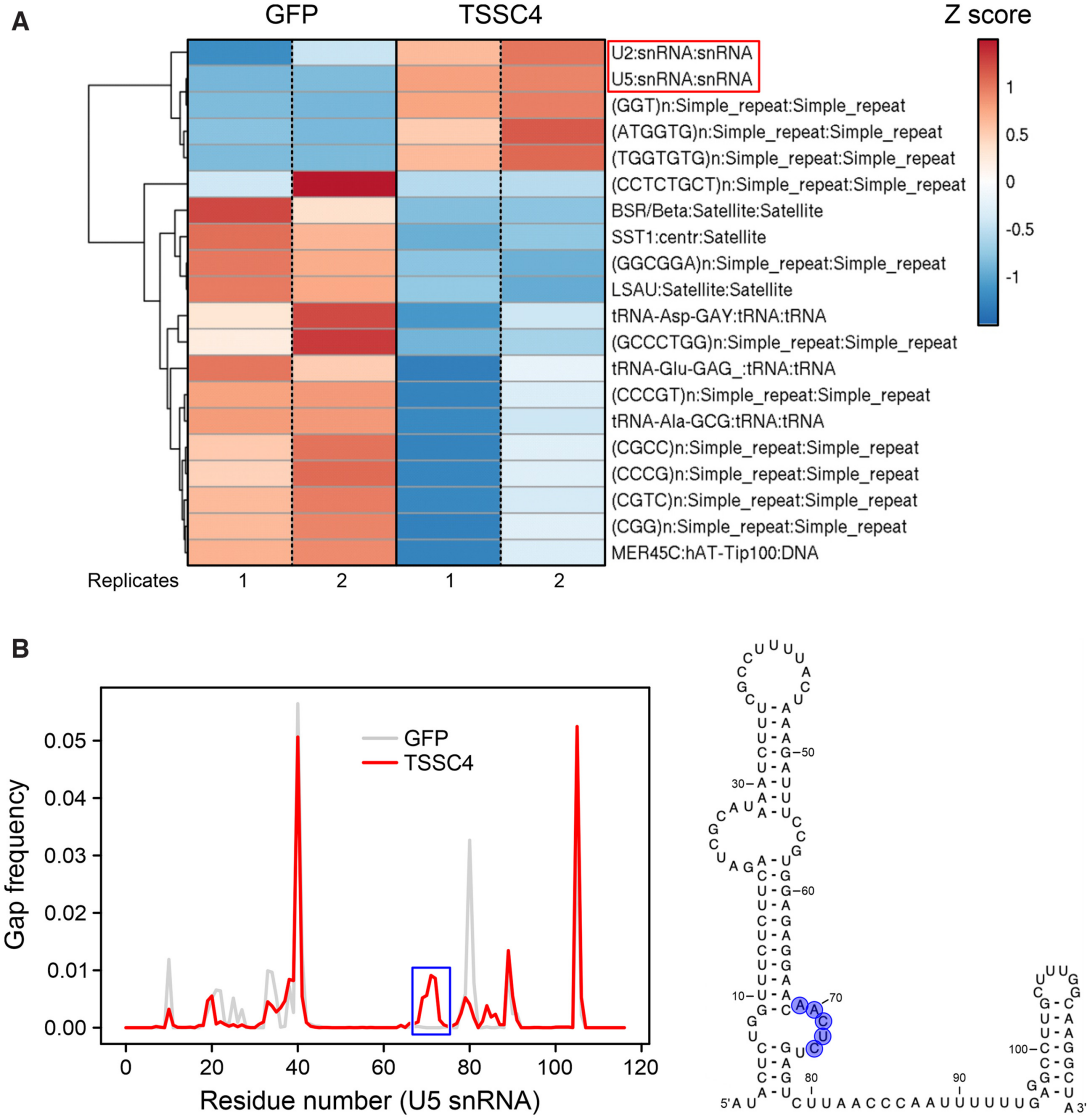
TSSC4 UV-crosslinks with U2 and U5 snRNA. (**A**) Heat map displaying the top 20 differentially enriched non-coding RNAs in GFP (negative control) and TSSC4 pull-down after RNA-protein UV-crosslinking and FLASH (44), analyzed with snakePipes (66). Two biological replicates each are shown. Colored by *Z*-score as indicated. Negatively enriched RNAs (blue) in the TSSC4 pull-down are mainly simple repeat RNAs and tRNAs that are commonly found as background in such analyses. (**B**) Left, gaps in sequencing reads for U5 snRNA from FLASH experiments with TSSC4 (red) or GFP (control; gray). Blue box, gap in reads for U5 snRNA nucleotides 69–73 in TSSC4-FLASH compared to GFP-FLASH. Right, putative TSSC4 crosslinking sites (blue background) mapped to a secondary structure model of U5 snRNA.

SnakePipes analysis (66) revealed significant TSSC4 crosslinking with U2 snRNA (log_2_-fold change = 1.48; *P*-value = 1.022575e–12) and U5 snRNA (log_2_-fold change = 4.97; *P*-value = 1.164354e–198; Figure 7A), implying intermittent, direct contacts of TSSC4 to these RNAs. While we did not observe unambiguous signatures identifying the residues of U2 snRNA crosslinked to TSSC4, FLASH data suggested direct contacts of TSSC4 to U5 snRNA residues 69–73 (Figure 7B). The observation of direct U5 snRNA contacts is consistent with the previous finding of TSSC4 and U5 snRNA in the same complexes by others (31). It is in line with TSSC4’s function as a U5 snRNP assembly/recycling factor and suggests that TSSC4 may help bring together and spatially align U5 snRNP-specific proteins and U5 snRNA (or Sm core RNPs) by also establishing transient, direct interactions with the RNA component. U2 and U5 snRNAs are both contained in post-splicing complexes (67), from which snRNPs need to be recycled for further rounds of splicing. We, therefore, suggest that direct interactions of TSSC4 with U2 snRNA might ensue during snRNP recycling, possibly to assist sorting of components to individual snRNPs.

## DISCUSSION

### Intrinsically disordered proteins in RNP biogenesis

Biogenesis of many RNPs in mammalian cells proceeds through specific pathways and involves specific assembly factors and chaperones. A prime example is afforded by ribosomal (r) subunits and ribosomes, whose assembly in eukaryotes requires over 350 non-ribosomal factors (68). While r-subunits continue to serve as major model systems for studies of RNP assembly mechanisms, their architecture is somewhat unusual compared to those of other cellular RNPs due to their sizes and compositional complexities, and as the subunit cores are made up predominantly of the long, compactly folded rRNAs, to which the numerous r-proteins need to be added at specific sites. Thus, many ribosome assembly factors are NTP-dependent RNA helicases, AAA-ATPases, ABC-ATPases, GTPases and kinases, which act, e.g., to remodel rRNAs/rRNPs, displace non-ribosomal factors, transport ribosomal components or surveil the quality of the emerging subunits (68–70). In contrast, many other RNPs, including snRNPs, harbor rather short RNA components and are comparatively protein-rich. Among the assembly factors controlling biogenesis of such RNPs, several intrinsically disordered proteins have been identified. For example, the IDP, NUFIP, has been shown to tether RNP components and other assembly machinery during biogenesis of box C/D and H/ACA small nucleolar RNPs, U4 snRNP, telomerase and selenoprotein-encoding mRNPs (71,72). However, how such IDPs support scaffolding functions on the molecular level and whether they serve additional specific functions during RNP assembly, for which their intrinsic disorder plays a role, remains largely unknown. Here, we have addressed such questions for the intrinsically disordered TSSC4 protein.

### Importance of the observed direct TSSC4–PRPF8/SNRNP200 interactions for snRNP assembly and recycling

Our analyses revealed TSSC4 interactions with snRNP proteins, other spliceosomal proteins as well as snRNP assembly factors and chaperones, which are dependent on (lost/reduced in the TSSC4^V3^-Flag-IP) or independent of (maintained in the TSSC4^V3^-Flag-IP) TSSC4’s interactions with the PRPF8 Jab1 domain and SNRNP200 CC. The latter type of interactions could involve additional direct contacts of TSSC4 to other proteins. Binding regions for other snRNP components, splicing factors and/or assembly factors and chaperones may be located within the ∼150 N-terminal residues of TSSC4 as well as the regions intervening between or following the PRPF8^Jab1ΔC^/SNRNP200^HR^-binding regions, which remain available in our cryoEM structure. Putative additional TSSC4 interactors suggested by structural comparisons are consistent with the observation that deletion of TSSC4 residues 51–100 or 75–100, or alanine mutagenesis in the region 85–100, although not affecting any of the PRPF8/SNRNP200-binding regions delineated here, have been observed to reduce co-precipitation with U5 snRNP components, including all subunits of the PRPF8–SNRNP200–EFTUD2–SNRNP40 sub-module (31). Furthermore, the previous observations that TSSC4 variants lacking residues 200–230, including SNRNP200-binding region 2, or bearing alanine exchanges of residues 213–218, affecting region 2, lead to dysfunctional U4/U6-U5 assembly (31) strongly support the notion that the direct interactions of TSSC4 with PRPF8 and SNRNP200 delineated here are important for the snRNP assembly and recycling processes that TSSC4 has been implicated in.

### Intrinsic disorder allows TSSC4 to bridge large distances

The observed TSSC4-binding sites are widely separated on the SNRNP200^HR^–PRPF8^Jab1ΔC^ complex, yet TSSC4 manages their concomitant occupation by employing only its C-terminal half. Clearly, the intrinsic disorder of TSSC4 is required to bridge between these binding sites while employing only a limited-length peptide region. Previously, deletion and point mutagenesis affecting TSSC4, in combination with GST pull-down assays, were employed to probe TSSC4 interactions *in cellulo* (31). Notably, while these studies did not distinguish direct and indirect interactions of TSSC4, the results can be fully reconciled by our structural analyses. Deletion of TSSC4 residues 75–175, removing PRPF8^Jab1^-binding region 1 (residues 153–168) but leaving SNRNP200-binding regions 2–4 intact, led to a reduction of TSSC4’s interaction with all tested U5 snRNP proteins, except SNRNP200 (31). Deletion of TSSC4 residues 201–250 or 200–230, or alanine exchanges of TSSC4 residues 213–218, which remove or alter SNRNP200^HR^-binding region 2 (residues 198–220), led to reduced co-precipitation of PRPF19 complex components, PRPF8 and/or SNRNP200 (31). While reduced SNRNP200 co-precipitation is explained by the disruption or alteration of a direct TSSC4-SNRNP200 contact, effects on PRPF8 and PRPF19 complex proteins are most likely indirect. As revealed by our structural analysis, the TSSC4 residue 201–250 or residue 200–230 deletion variants not only lead to removal of an SNRNP200-binding region but also probably give rise to a binding conflict; reducing the spacer between PRPF8^Jab1^-binding region 1 and SNRNP200-binding region 3 by 50 or 31 residues may no longer allow TSSC4 to bridge the corresponding distances on the SNRNP200–PRPF8^Jab1^ complex. Reduced co-precipitation of PRPF19 complex components *via* the TSSC4 deletion variants might reflect a disturbed TSSC4 association with post-splicing complexes *via* PRPF8 and SNRNP200 for snRNP recycling.

### TSSC4 intrinsic disorder may support PRPF8-SNRNP200 complex formation and U5 snRNP sub-module assembly

Upon interacting with a folded protein or protein domain, IDPs preclude slow association rates as they are not subject to orientation restraints that may govern the interaction of two folded proteins (73). Thus, while the SNRNP200^HR^–PRPF8^Jab1^ interaction is thermodynamically comparatively stable (74), it may be kinetically controlled *in vivo*. Fast and concomitant interaction of TSSC4 with both components may, thus, increase the speed of PRPF8-SNRNP200 complex formation in cells. This notion is further supported by our observation that the TSSC4 binding regions do not adopt regular secondary structures upon binding, suggesting that no potentially rate-limiting folding step is involved in the binding mechanism.

Furthermore, the PRPF8^Jab1ΔC^-binding region 1 and the most N-terminal SNRNP200^HR^-binding region 2 on TSSC4 are separated by about 30 residues, which will limit the relative distance between the SNRNP200 CC and PRPF8 Jab1 domain once they are both engaged by TSSC4. TSSC4 has been shown to exist in U5 snRNP or U4/U6–U5 tri-snRNP assembly intermediates together with other assembly factors, including AAR2 (26,31). Our comparison of a putative, yeast-like PRPF8–AAR2 complex to the SNRNP200^HR^–PRPF8^Jab1ΔC^-TSSC4 cryoEM structure suggests that TSSC4 may help to capture the PRPF8 Jab1 domain from an AAR2–PRPF8 complex (possibly also containing PRPF8-bound EFTUD2 and SNRNP40), restrict its spatial orientation relative to SNRNP200 and, by fostering formation of the PRPF8^Jab1^–SNRNP200 interaction and precluding alternative interactions of PRPF8^Jab1^ on the AAR2-PRPF8 intermediate, guide assembly of the PRPF8–SNRNP200–EFTUD2–SNRNP40 sub-module.

### TSSC4 may suppress aberrant SNRNP200 helicase activity during snRNP assembly

RNA helicases, such as SNRNP200, usually can unwind RNAs or disrupt RNPs without an apparent sequence specificity. Already during formation of U5 snRNP and of the U4/U6–U5 tri-snRNP, SNRNP200 is incorporated into the same particles as U5 snRNA and the U4/U6 di-snRNA, its cognate substrate. Thus, during U5 snRNP and U4/U6–U5 tri-snRNP assembly, and before SNRNP200 is stably anchored away from U4/U6 di-snRNA as seen in the mature human tri-snRNP (51,75), the SNRNP200 RNA helicase activity may need to be controlled to prevent remodeling of non-cognate RNAs/RNPs, such as partly protein-decorated U5 snRNA, or unwanted disruption of pre-mature U4/U6 di-snRNP. While some of this control may be exerted by the SNRNP200-inhibitory activity of the PRPF8 Jab1 domain, our analyses suggest that TSSC4 reinforces SNRNP200 inhibition during snRNP assembly. Interestingly, TSSC4 can exert this control while predominantly contacting the inactive SNRNP200 CC. Allosteric inhibition of the SNRNP200/Brr2p helicase activity by CC-binding IDPs has been observed before for the FBP21 B-specific protein in human (34) and the NTR2 protein in yeast (76), and may involve restriction of functional NC-CC flexibility. In the case of TSSC4, its additional contacts to the PRPF8 Jab1 domain may reinforce the insertion of the Jab1 domain's inhibitory C-terminal tail into the SNRNP200 RNA-binding tunnel.

### TSSC4 acts as a placeholder during snRNP assembly

The profound conformational and compositional dynamics that the spliceosome undergoes during its duty cycle, which involve the repeated recruitment and release of splicing factors sometimes employing common binding sites on core spliceosomal subunits (1,3,4), poses a fundamental problem for snRNP assembly - how can interactions that are to ensue only during splicing be prevented during the initial assembly or during recycling of the basic building blocks? Part of the solution to this problem may be the compartmentalization of processes, with active splicing taking place predominantly in peri-chromatin fibrils (77,78) and snRNP assembly/recycling taking place in Cajal bodies (14,15). An additional contribution, exemplified by our study of TSSC4, may be snRNP assembly factors that act as placeholders, transiently occupying functionally important sites on core splicing factors. Comparison of our SNRNP200^HR^–PRPF8^Jab1ΔC^–TSSC4 cryoEM structure to structures of other spliceosomal complexes containing SNRNP200 and PRPF8 showed that TSSC4 regions transiently block binding sites on SNRNP200, which also serve as attachment points for constitutive splicing factors (PRPF28, FBP21) and splicing regulatory proteins (C9ORF78) during splicing.

Placeholders have previously been identified as factors involved in ribosome biogenesis, where they seem to avoid folding traps or act as timers for surveillance processes during assembly (79). In addition to such functions, place-holding by TSSC4 during snRNP assembly may also help to prevent incorporation of U4/U6-U5 tri-snRNP assembly intermediates into spliceosomes, which may otherwise compete with mature U4/U6–U5 tri-snRNP for incorporation into the pre-catalytic B complex, and/or to prevent pre-mature association of splicing factors (such as FBP21 or C9ORF78) with U4/U6–U5 tri-snRNP assembly intermediates. Thereby, TSSC4 would help preserve the spliceosome's full regulatory capacity (as represented in this case by unoccupied binding sites on SNRNP200) at the beginning of a splicing cycle. In line with this function, our competitive binding assays suggest a higher affinity of TSSC4 for SNRNP200 as compared to FBP21 or C9ORF78.

### Comparative TSSC4 interactome studies suggest multiple TSSC4-dependent assembly/recycling stages

Interestingly, we found a striking pattern when comparing interactions of TSSC4^wt^ and TSSC4^V3^ with different groups of snRNP assembly factors and chaperones. TSSC4^V3^, lacking stable PRPF8^Jab1^/SNRNP200^HR^ binding *in vitro*, exhibited enhanced interactions with all components of the SMN complex. In contrast, TSSC4 interactions with U5 snRNP assembly factors were largely PRPF8^Jab1^/SNRNP200^HR^-independent, while co-precipitation of snRNP assembly chaperone machinery was severely reduced for TSSC4^V3^ compared to TSSC4^wt^. While a detailed understanding of these differences will require additional analyses, our findings are consistent with several distinct TSSC4-dependent phases or steps during U5 snRNP or U4/U6–U5 tri-snRNP assembly. For example, TSSC4 together with other U5 snRNP assembly factors, such as AAR2, may give rise to the U5 tetrameric sub-module (PRPF8–SNRNP200–EFTUD2–SNRNP40), which might then be further matured, and perhaps expanded to a larger pre-U5 particle (or 20S U5 snRNP), with the help of snRNP assembly chaperones. Disrupting TSSC4 interactions with PRPF8 and SNRNP200 may stall the process at a stage before assembly chaperones function (reduced interaction of TSSC4^V3^ with snRNP assembly chaperones). The interaction of TSSC4 with SMN complex components likely reflects the involvement of Cajal bodies in TSSC4-dependent U5 assembly steps. Stalling the assembly process by disrupting TSSC4–PRPF8/SNRNP200 contacts may trap TSSC4-bound assembly intermediates in or on Cajal bodies (enhanced interaction of TSSC4^V3^ with SMN complex subunits). The above scenario is consistent with the observation that TSSC4 down-regulation increases U5 snRNA accumulation in Cajal bodies (31), the same effect that is caused by PRPF8 down-regulation (18). In any case, our TSSC4^V3^ variant may constitute a useful tool to further dissect individual steps along the U5 snRNP assembly pathway.

### TSSC4-dependent snRNP recycling

As in human the U5 snRNP is remodeled during splicing and becomes tightly associated with the PRPF19 complex and additional proteins (17), our observation of TSSC4 interacting with PRPF19-related proteins supports an additional function of TSSC4 during post-splicing U5 snRNP recycling, as suggested previously (31). TSSC4-binding sites on SNRNP200 and the PRPF8 Jab1 domain, as detected here, are unoccupied in the human post-splicing P complex (PDB ID 6QDV) (67), such that TSSC4 could latch onto a post-catalytic spliceosome to initiate recycling. This model might explain the B^act^ and C complex proteins that we detected as interactors of TSSC4^wt^, but for the most part not of TSSC4^V3^, as most of these proteins are contained in the human P complex (except B^act^ complex protein PPIL2 and C complex protein DDX35) (80). Moreover, it has been suggested that aberrant spliceosomal intermediates can be re-routed to discard pathways (81,82), such that snRNPs may also need to be recycled from various discarded complexes with diverse subunit compositions, in the case of U5 snRNP likely aided by TSSC4. Finally, participation of TSSC4 in recycling U5 snRNP from post-splicing or discarded complexes may also explain our observation of TSSC4 interactions with U2 snRNA, which is contained in the post-catalytic spliceosome (67).

### Its intrinsic disorder may facilitate TSSC4 displacement during late assembly stages

Ultimately, TSSC4 needs to be displaced to give rise to mature U5 snRNP or U4/U6–U5 tri-snRNP. Entropy-enthalpy compensation has been suggested as a fundamental principle underlying the formation of highly specific IDP–protein interactions that still exhibit limited thermodynamic stability and are, thus, reversible (83). This principle also seems to be realized by TSSC4. Each TSSC4 binding region buries a considerable amount of surface area upon interaction with PRPF8^Jab1ΔC^ or SNRNP200^HR^ (interface areas between 526 and 895 Å^2^), suggesting that the individual regions are recognized with high specificity. At the same time, we observe that the TSSC4 regions become immobilized upon binding, as revealed by clearly defined cryoEM density. This observation suggests that complex formation may be associated with a significant loss of conformational entropy within the bound TSSC4 regions. We, therefore, suggest that the highly specific interactions that ensue between TSSC4 regions and the SNRNP200^HR^–PRPF8^Jab1ΔC^ complex are still of limited thermodynamic stability, as would be required for an assembly factor that relies on specific, yet reversible binding.

Comparison of our SNRNP200^HR^–PRPF8^Jab1ΔC^–TSSC4 cryoEM structure to the structure of a U4/U6–U5 tri-snRNP revealed minor but noticeable steric conflicts. Thus, specific interactions among U4/U6–U5 tri-snRNP components may outcompete TSSC4 during late stages of U4/U6–U5 tri-snRNP assembly. The formation of multiple independent, spatially separated PRPF8/SNRNP200 contacts *via* discontinuous TSSC4 regions, each of limited thermodynamic stability, as opposed to the formation of a continuous binding surface, may be a principle that supports the stepwise removal of TSSC4 during late stages of U4/U6–U5 tri-snRNP assembly.

## DATA AVAILABILITY

CryoEM reconstructions have been deposited at the Electron Microscopy Data Bank (https://www.ebi.ac.uk/pdbe/emdb) (84) under accession code EMD-13690 (https://www.ebi.ac.uk/pdbe/entry/emdb/EMD-13690). Structure coordinates have been deposited at the RCSB Protein Data Bank (https://www.rcsb.org) (85) under accession code 7PX3 (https://doi.org/10.2210/pdb7PX3/pdb). Mass spectrometry proteomics data have been deposited at the ProteomeXchange Consortium *via* the PRIDE partner repository (86) with the dataset identifier PXD029493. FLASH data have been deposited at Gene Expression Omnibus (https://www.ncbi.nlm.nih.gov/geo/) (87) under accession code GSE185418 (https://www.ncbi.nlm.nih.gov/geo/query/acc.cgi?acc=GSE185418). All other data are contained in the manuscript or the supplemental material. Materials are available from the corresponding author upon reasonable request.

## Supplementary Material

gkac087_Supplemental_Files

## Notes

Present address: Alexandra Bergfort, Yale University, Molecular Biophysics and Biochemistry, 333 Cedar Street, New Haven, CT 06510, USA.
