# Supplementary material for: The intrinsically disordered TSSC4 protein acts as a helicase inhibitor, placeholder and multi-interaction coordinator during snRNP assembly and recycling
Source: Nucleic Acids Res. 2022 Feb 21;50(5):2938–58. doi: 10.1093/nar/gkac087 (PMC8934646; doi:10.1093/nar/gkac087)
Supplement: gkac087_Supplemental_Files [file gkac087_supplemental_files.zip › Supplement revised, 17-01-2022.pdf]

SUPPLEMENTARY DATA

The intrinsically disordered TSSC4 protein acts as a helicase inhibitor, placeholder and multi-interaction coordinator during snRNP assembly and recycling

Alexandra Bergfort[1], Tarek Hilal[1,2], Benno Kuropka[3,4], İbrahim Avşar Ilik[5], Gert Weber[6], Tuğçe Aktaş[5], Christian Freund[3], Markus C. Wahl[1,6,*]

[1] Freie Universität Berlin, Institute of Chemistry and Biochemistry, Laboratory of Structural Biochemistry, Takustr. 6, D-14195 Berlin, Germany

[2] Freie Universität Berlin, Institute of Chemistry and Biochemistry, Research Center of Electron Microscopy, Fabeckstr. 36a, 14195 Berlin, Germany

[3] Freie Universität Berlin, Institute of Chemistry and Biochemistry, Laboratory of Protein Biochemistry, Thielallee 63, D-14195, Berlin, Germany

[4] Freie Universität Berlin, Institute of Chemistry and Biochemistry, Core Facility BioSupraMol, Thielallee 63, D-14195, Berlin, Germany

[5] Max Planck Institute for Molecular Genetics, Ihnestr. 63, D-14195 Berlin, Germany

[6] Helmholtz-Zentrum Berlin für Materialien und Energie, Macromolecular Crystallography, Albert-Einstein-Str. 15, D-12489 Berlin, Germany

* Correspondence to: markus.wahl@fu-berlin.de

SUPPLEMENTARY TABLES

Supplementary Table S1. CryoEM data collection and refinement

Complex	SNRNP200^{HR}-PRPF8$^{Jab1\Delta C}$-TSSC4
Microscope	FEI Titan Krios G3i
Voltage [keV]	300
Camera	Falcon 3EC
Magnification	120,000
Pixel size [Å/pixel]	0.657
Total electron exposure [e^-/Å^2]	40
Exposure rate [e^-/pixel/s]	0.6
No. of frames collected during exposure	33
Defocus range [µm]	0.8 - 1.8
Automation software	EPU2.10
Micrographs collected	3,566
Micrographs used	3,349
Total extracted particles	1,025,529
Refined particles	611,449
Final particles	387,973
Point group or helical symmetry parameters	C1
$FSC_{0.143}$ (unmasked / masked) [Å]	3.3 / 3.05
Global resolution [Å]	3.05
Local resolution range [Å]	2.46 - 30.0
Map sharpening B factor [Å^2]	-105
Map sharpening method	Local B factor
CC mask	0.88
CC volume	0.87
Model<br>Non-H atoms<br>Protein residues	 16,426<br>2,041
RMSD[1]<br>Bond lengths [Å]<br>Bond angles [°]	 0.003<br>0.524
Ramachandran plot<br>Favored [%]<br>Allowed [%]<br>Outliers [%]	 97.63<br>2.37<br>0
Model quality[2]<br>Clash score<br>Rotamer outliers [%]<br>Overall score	 7.20<br>5.20<br>2.02
EMDB ID	EMD-13690
PDB ID	7PX3

1 RMSD, root-mean-square deviation from ideal geometry
2 Assessed using MolProbity (1)

Supplementary Table S2. Results from TSSC4^{wt}-Flag IP and TSSC4^{V3}-Flag IP

See attached Excel file.

SUPPLEMENTARY FIGURES

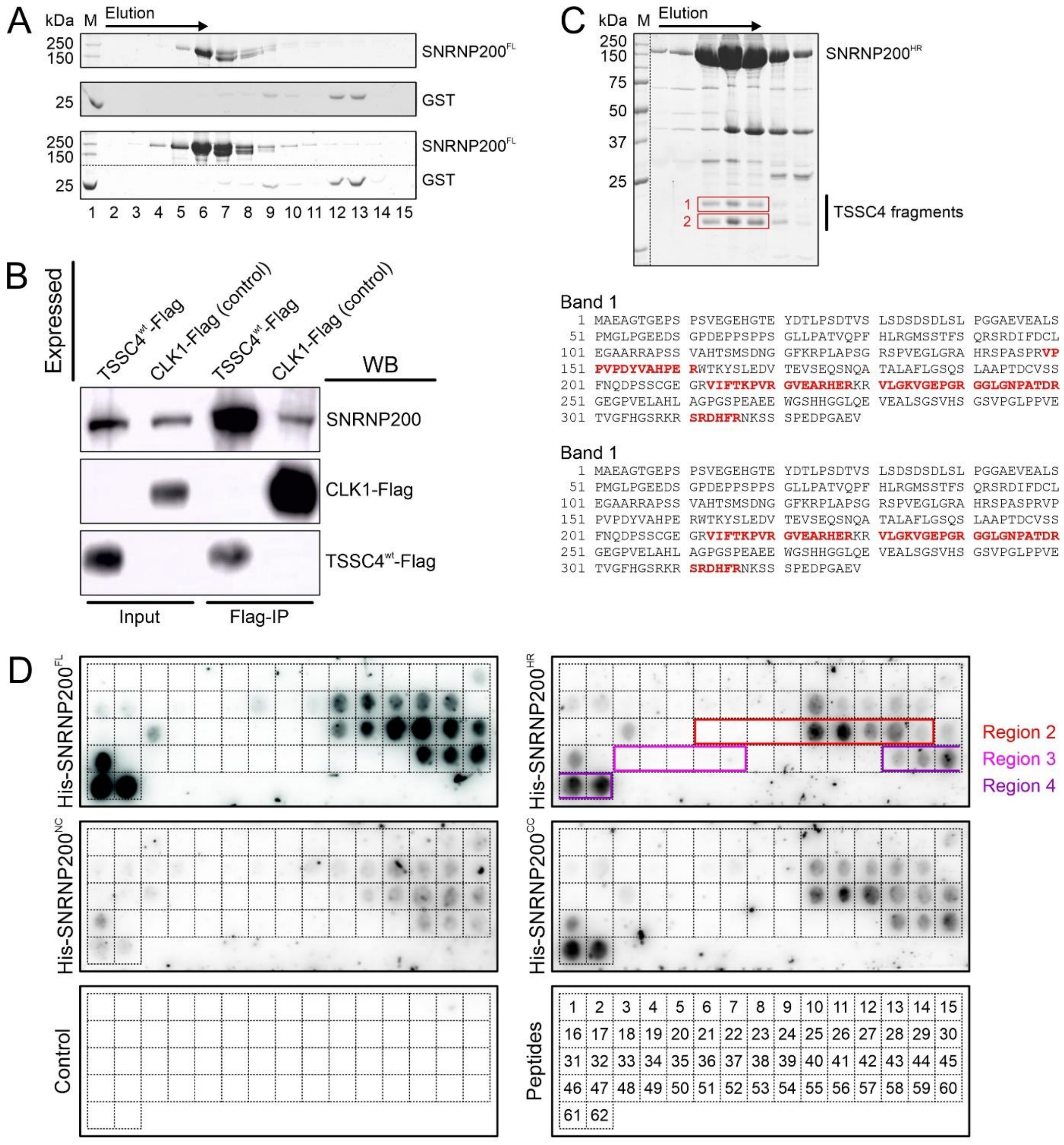

Peptide	TSSC4 residues
1	1-25
2	6-30
...	...
61	301-325
62	306-329

Supplementary Figure S1. SNRNP200-TSSC4 interaction studies

(**A**) SDS-PAGE analysis of elution fractions from analytical SEC, monitoring interaction of GST with SNRNP200^{FL} (negative control). The elution direction is indicated by an arrow above the gels. All runs were conducted under identical conditions and the same elution fractions are shown for each run. Protein bands are identified on the right. M, molecular mass marker. In the third panel, upper and lower regions of the same gel were spliced together for clarity. Dotted line, splice position. (**B**) Flag-IP from nuclear extract of HEK293 cells expressing TSSC4^{wt}-Flag or CLK1-Flag (negative control) followed by Western blot (WB) using an anti-SNRNP200 antibody (top) or an anti-Flag antibody (middle and bottom). Protein bands are identified on the right. (**C**) Top, SDS-PAGE analysis of elution fractions from analytical SEC of a SNRNP200^{HR}-GST-TSSC4 complex after treatment with chymotrypsin. The elution direction is indicated by an arrow above the gels. Protein bands are identified on the right. M, molecular mass marker. Gel bands subjected to mass spectrometric analysis are highlighted by red boxes and numbered. Bottom, TSSC4 peptides identified in the analyzed gel bands by mass spectrometry. Identified peptides, bold red. (**D**) TSSC4 peptide SPOT arrays incubated with the His-tagged SNRNP200 constructs indicated on the left (control, no protein) and probed using an anti-His antibody. Lower right panel, peptide spotting scheme; table on the bottom, TSSC4 residues contained in each peptide; colored boxes in the array probed with His-SNRNP200^{HR}, peptides containing at least 5 residues of TSSC4 regions 2-4 that contact SNRNP200^{HR} in the SNRNP200^{HR}-PRPF8$^{Jab1\Delta C}$-TSSC4 cryoEM structure. A putative weak binding region represented by peptides 25-29 (covering TSSC4 residues 121-165) does not coincide with an SNRNP200^{HR}-binding TSSC4 region in our structural analysis.

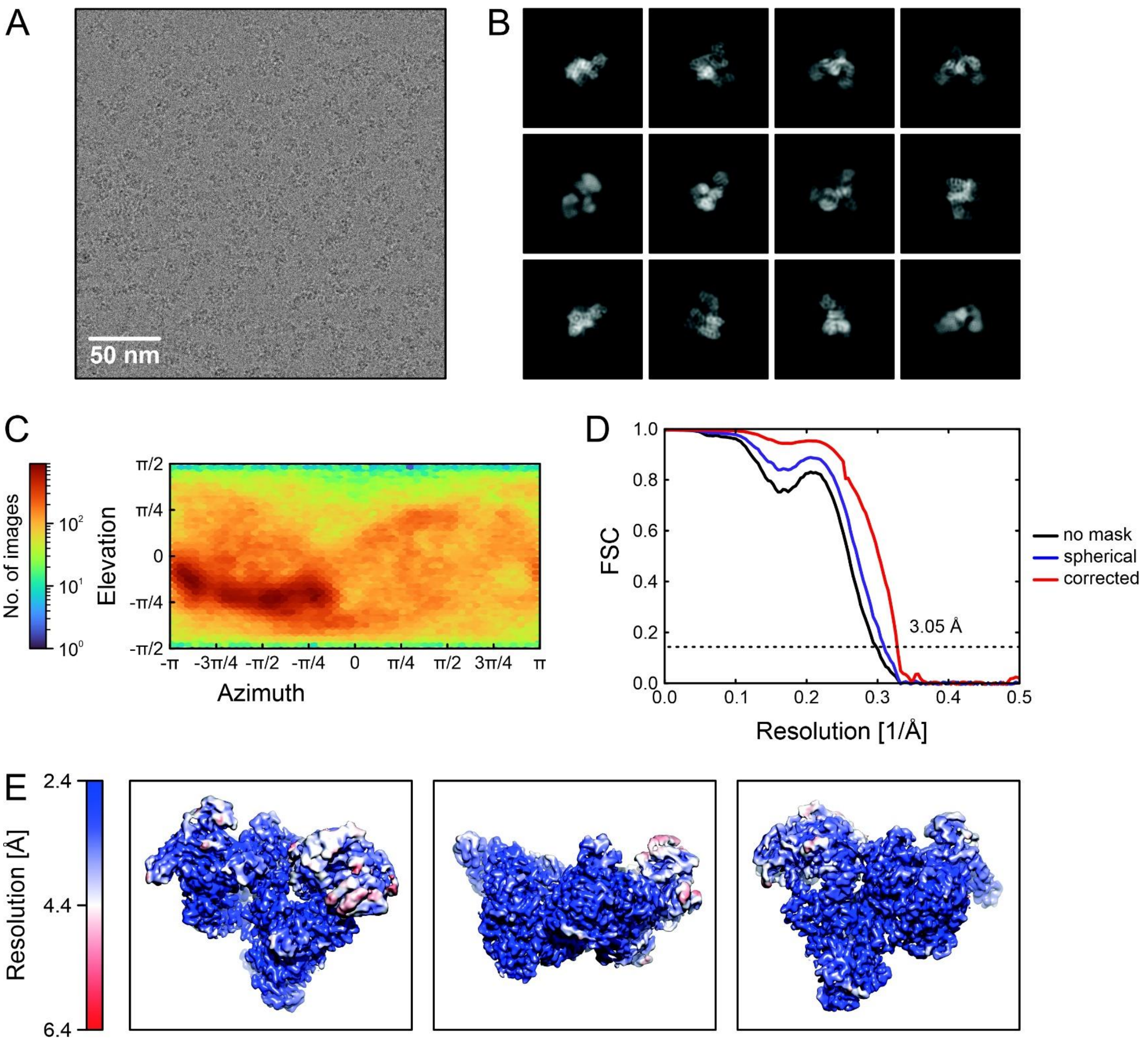

Supplementary Figure S2. CryoEM analysis

(**A**) Representative cryoEM micrograph of the SNRNP200^{HR}-PRPF8$^{Jab1\Delta C}$-TSSC4 complex. Scale bar, 50 nm. (**B**) Class averages of SNRNP200^{HR}-PRPF8$^{Jab1\Delta C}$-TSSC4 particle images after reference-free 2D classification. (**C**) Orientation distribution plot of the particle images used for the final SNRNP200^{HR}-PRPF8$^{Jab1\Delta C}$-TSSC4 cryoEM reconstruction, as obtained during non-uniform refinement with cryoSPARC. (**D**) Global resolution estimation for the SNRNP200^{HR}-PRPF8$^{Jab1\Delta C}$-TSSC4 cryoEM reconstruction by gold standard Fourier shell correlation (FSC).

Dashed line, $FSC_{0.143}$. (**E**) Local resolution estimation as determined with cryoSPARC, ranging from 2.46 Å to 38 Å for the $SNRNP200^{HR}$-$PRPF8^{Jab1\Delta C}$-TSSC4 cryoEM reconstruction.

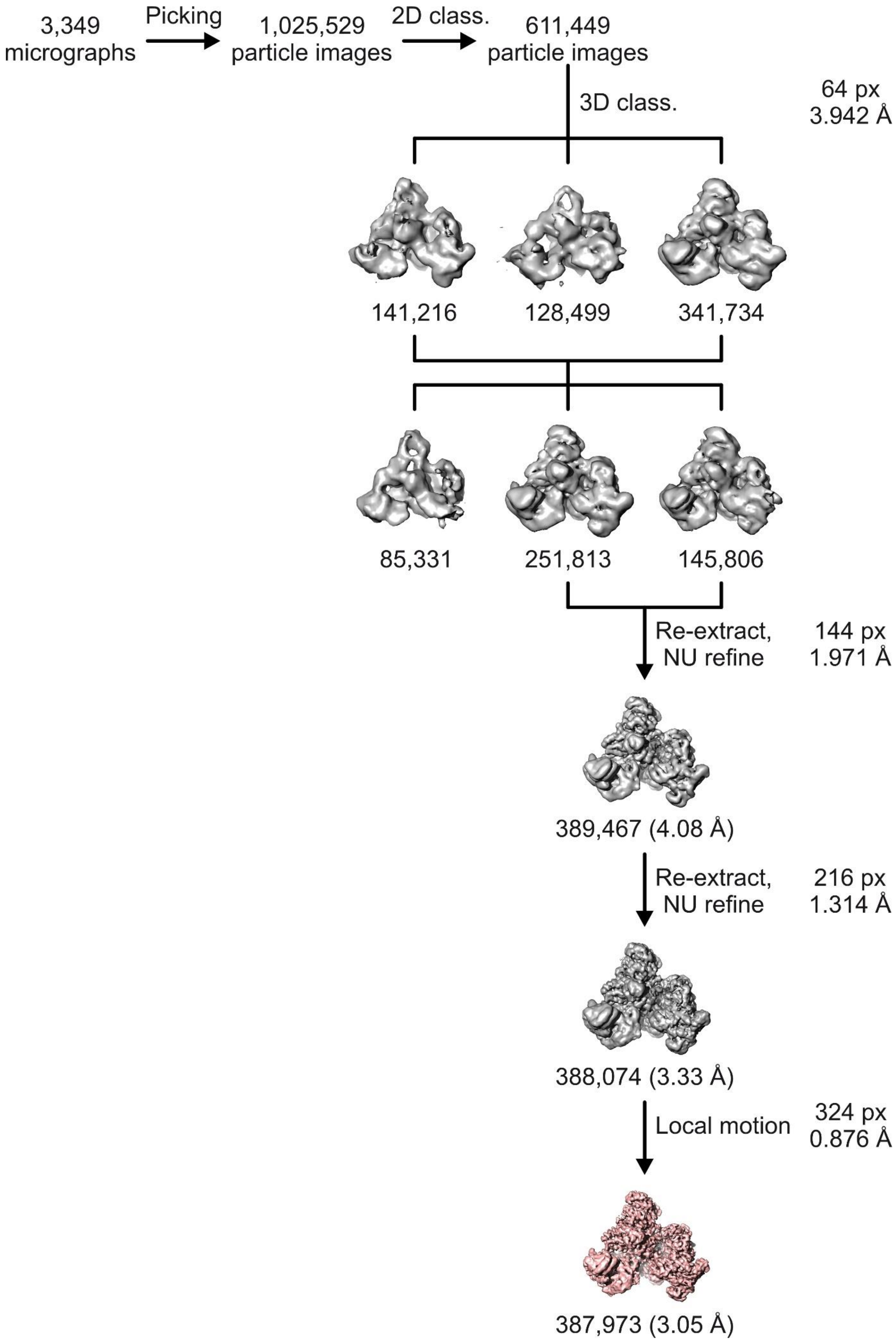
3,349 micrographs
Picking
1,025,529 particle images
2D class.
611,449 particle images
3D class.
64 px
3.942 Å
141,216
128,499
341,734
85,331
251,813
145,806
Re-extract, NU refine
144 px
1.971 Å
389,467 (4.08 Å)
Re-extract, NU refine
216 px
1.314 Å
388,074 (3.33 Å)
Local motion
324 px
0.876 Å
387,973 (3.05 Å)

Supplementary Figure S3. CryoEM reconstruction

Scheme illustrating the cryoEM reconstruction strategy. 1,025,529 particle images were initially picked from 3,349 micrographs, extracted with a box size of 384 px, Fourier-cropped to 64 px and subjected to reference-free 2D classification. 611,449 particle images were selected for iterative cycles of heterogeneous 3D refinements, after which 389,467 particles were re-extracted with a box size of 432 px, Fourier-cropped to 144 px and refined by non-uniform (NU) refinement to a resolution of 4.08 Å. After re-extraction with Fourier-cropping to a box of 216 px, NU refinement resulted in a 3.33 Å reconstruction. As CTF refinement did not improve the resolution, local motion correction was applied to generate Fourier-cropped particle images with a pixel size of 0.876 Å (box size 324 px). The final reconstruction was generated by NU refinement, yielding a global resolution of 3.05 Å.

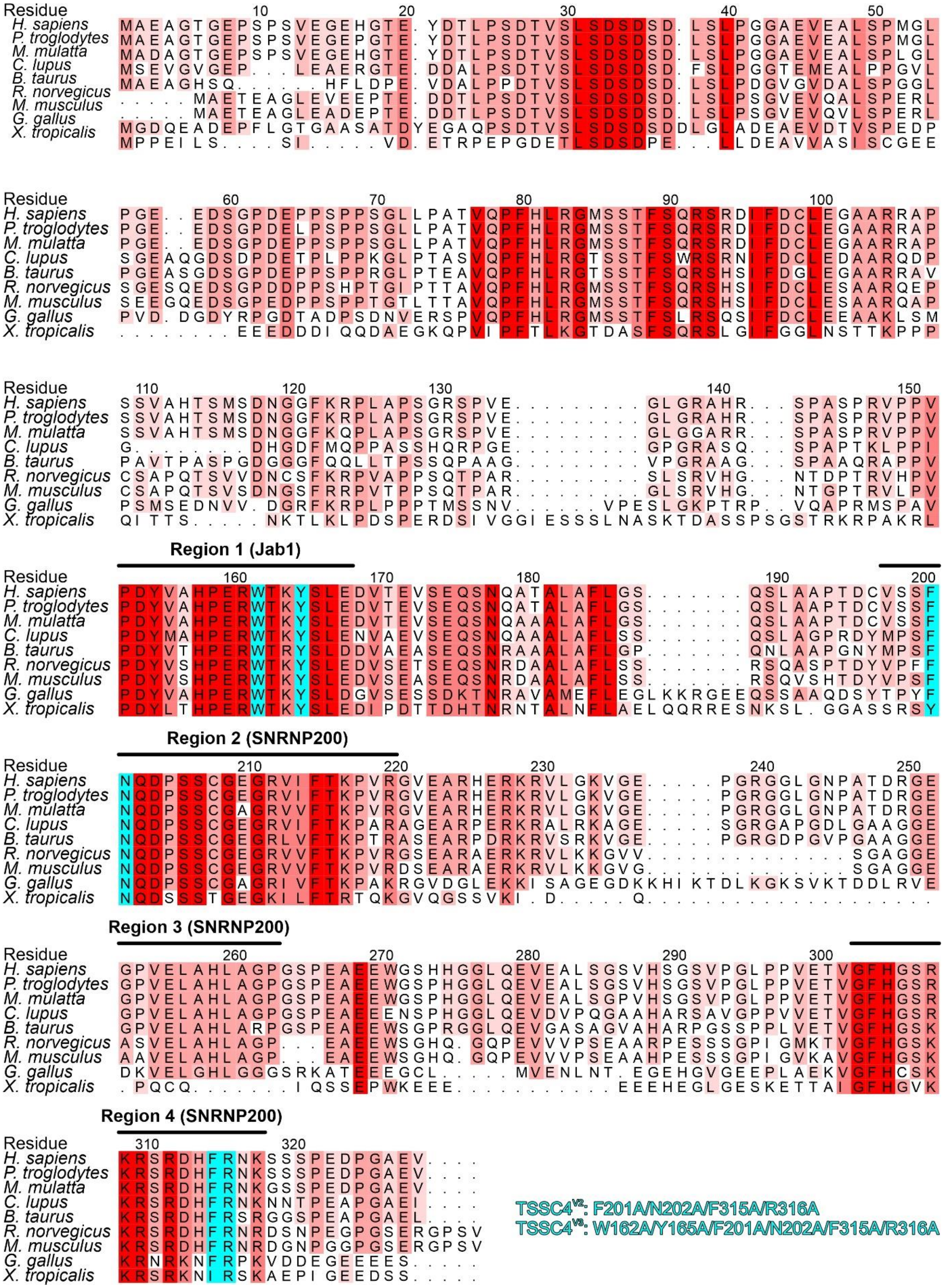
Residue 10 20 30 40 50
H. sapiens MAEAGTGEPSPSVEGEHGTE.YDTLPSDTVSLSDSDSD.LSLPGGAEVEALSPMGL
P. troglodytes MAEAGTGEPSPSVEGEPGTE.YDTLPSDTVSLSDSDSD.LSLPGGAEVEALSPMGL
M. mulatta MADAGTGEPSPSVEGEHGTE.YDTLPSDTVSLSDSDSD.LSLPGGAEVEALSPLGL
C. lupus MSEVGVGEP...LEAERGTE.DDALPSDTVSLSDSDSD.FSLPGGTEMEALPPGVL
B. taurus MAEAGHSQ......HFLDPE.VDALPPDTVSLSDSDSD.LSLPDGVGVDALSPGGL
R. norvegicus .....MAETEAGLEVEEPTE.DDTLPSDTVSLSDSDSD.LSLPSGVEVQALSPERL
M. musculus .....MAETEAGLEADEPTE.DDTLPSDTVSLSDSDSD.LSLPSGVEVQVLSPERL
G. gallus MGDQEADEPFLGTGAASATDYEGAQPSDTVSLSDSDSDDLGLADEAEVDTVSPEDP
X. tropicalis MPPEILS....SI.....VD.ETRPEPGDETLSDSDPE...LLDEAVVASISCGEE
Residue 60 70 80 90 100
H. sapiens PGE..EDSGPDEPPSPPSGLLPATVQPFHLRGMSSTFSQRSRDIFDCLEGAARRAP
P. troglodytes PGE..EDSGPDELPSPPSGLLPATVQPFHLRGMSSTFSQRSRDIFDCLEGAARRAP
M. mulatta PGE..EDSGPDEPPSPPSGLLPATVQPFHLRGMSSTFSQRSRDIFDCLEGAARRAP
C. lupus SGEAQGDSDPDETPLPPKGLPTASVQPFHLRGTSSTFSWRSRNIFDCLEDAARQDP
B. taurus PGEASGDSGPDEPPSPPRGLPTEAVQPFHLRGTSSTFSQRSHSIFDGLEGAARRAV
R. norvegicus SGESQEDSGPDDPPSHPTGIPTTAVQPFHLRGMSSTFSQRSHSIFDCLESAARQEP
M. musculus SEEGQEDSGPEDPPSPPTGTLTTAVQPFHLRGMSSTFSQRSHSIFDCLESAARQAP
G. gallus PVD.DGDYRPGDTADPSDNVERSPVQPFHLRGMSSTFSLRSQSIFDCLEEAAKLSM
X. tropicalis ........EEEDDDIQQDAEGKQPVIPFTLKGTDASFSQRSLGIFGGLNSTTKPPP
Residue 110 120 130 140 150
H. sapiens SSVAHTSMSDNGGFKRPLAPSGRSPVE.........GLGRAHR...SPASPRVPPV
P. troglodytes SSVAHTSMSDNGGFKRPLAPSGRSPVE.........GLGRAHR...SPASPRVPPV
M. mulatta SSVAHTSMSDNGGFKQPLAPSGRSPVE.........GLGGARR...SPASPRVPPV
C. lupus G........DHGDFMQPPASSHQRPGE.........GPGRASQ...SPAPTKLPPI
B. taurus PAVTPASPGDGGGFQQLLTPSSQPAAG.........VPGRAAG...SPAAQRAPPV
R. norvegicus CSAPQTSVVDNCSFKRPVAPPSQTPAR.........SLSRVHG...NTDPTRVHPV
M. musculus CSAPQTSVSDNGSFRRPVTPPSQTPAR.........GLSRVHG...NTGPTRVLPV
G. gallus PSMSEDNVV.DGRFKRPLPPPTMSSNV.....VPESLGKPTRP..VQAPRMSPAV
X. tropicalis QITTS.....NKTLKLPDSPERDSIVGGIESSSLNASKTDASSPSGSTRKRPAKRL
Region 1 (Jab1)
Residue 160 170 180 190 200
H. sapiens PDYVAHPERWTKYSLEDVTEVSEQSNQATALAFLGS.......QSLAAPTDCVSSF
P. troglodytes PDYVAHPERWTKYSLEDVTEVSEQSNQATALAFLGS.......QSLAAPTDCVSSF
M. mulatta PDYVAHPERWTKYSLEDVTEVSEQSNQAAALAFLGS.......QSLAAPTDCVSSF
C. lupus PDYMAHPERWTKYSLENVAEVSEQSNQAAALAFLSS.......QSLAGPRDYMPSF
B. taurus PDYVTHPERWTRYSLDDVAEASEQSNRAAALAFLGP.......QNLAAPGNYMPSF
R. norvegicus PDYVSHPERWTKYSLEDVSETSEQSNRDAALAFLSS.......RSQASPTDYVPFF
M. musculus PDYVSHPERWTKYSLEDVSEASEQSNRDAAMAFLSS.......RSQVSHTDYVPSF
G. gallus PDYVAHPERWTKYSLDGVSESSDKTNRAVAMEFLEGLKKRGEEQSSAAQDSYTPYF
X. tropicalis PDYLTHPERWTKYSLEDIPDTTDHTNRNTALNFLAELQQRRESNKSL.GGASSRSY
Region 2 (SNRNP200)
Residue 210 220 230 240 250
H. sapiens NQDPSSCGEGRVIFTKPVRGVEARHERKRVLGKVGE.....PGRGGLGNPATDRGE
P. troglodytes NQDPSSCGEGRVIFTKPVRGVEARHERKRVLGKVGE.....PGRGGLGNPATDRGE
M. mulatta NQDPSSCGAGRVVFTKPVRGVEARHERKRVLGKVGE.....PGRGGLGNPATDRGE
C. lupus NQDPSSCGEGRVIFTKPARAGEARPERKRALRKAGE.....SGRGAPGDLGAAGGE
B. taurus NQDPSSCGEGRLVFTKPTRASEARPDRKRVSRKVGE.....PGRGDPGVPGAAGGE
R. norvegicus NQDPSSCGEGRVVFTKPVRGSEARAERKRVLKKGVV...............SGAGGE
M. musculus NQDPSSCGEGRVVFTKPVRDSEARAERKRVLKKGVG...............SGAGGE
G. gallus NQDPSSCGAGRIVFTKPAKRGVDGLEKKISAGEGDKKHIKTDLKGKSVKTDDLRVE
X. tropicalis NQDSSSTGEGKILFTRTQKGVQGSSVKI.D.....Q....................
Region 3 (SNRNP200)
Residue 260 270 280 290 300
H. sapiens GPVELAHLAGPGSPEAEEWGSHHGGLQEVEALSGSVHSGSVPGLPPVETVGFHGSR
P. troglodytes GPVELAHLAGPGSPEAEEWGSPHGGLQEVEALSGSVHSGSVPGLPPVETVGFHGSR
M. mulatta GPVELAHLAGPGSPEAEEWGSPHGGLQEVEALSGPVHSGSVPGLPPVETVGFHGSR
C. lupus GPVELAHLAGPGSPEAEEENSPHGGLQEVDVPQGAAHARSAVGPPVVETVGFHGSR
B. taurus GPVELAHLARPGSPEAEEWSGPRGGLQEVGASAGVAHARPGSSPPLVETVGFHGSK
R. norvegicus ASVELAHLAGP...EAEEWSGHQ.GQPEVVVPSEAARPESSSGPIGMKTVGFHGSK
M. musculus AAVELAHLAGP...EAEEWSGHQ.GQPEVVVPSEAAHPESSSGPIGVKAVGFHGSK
G. gallus DKVELGHLGGGSRKATEEEGCL.....MVENLNT.EGEHGVGEEPLAEKVGFHCSK
X. tropicalis .PQCQ.......IQSSEPWKEEE..........EEEHEGLGESKETTAIGFHGVK
Region 4 (SNRNP200)
Residue 310 320
H. sapiens KRSRDHFRNKSSSPEDPGAEV....
P. troglodytes KRSRDHFRNKSSSPEDPGAEV....
M. mulatta KRSRDHFRNKGSSPEDPGAEV....
C. lupus KRSRDHFRNKNNTPEAPGAEI....
B. taurus KRSRDHFRSRGGSPEAPGAEL....
R. norvegicus KRSRDHFRNRDSNPEGPGSERGPSV
M. musculus KRSRDHFRNRDGNPGGPGSERGPSV
G. gallus KRNRKNFRPKVDDEGEEEES.....
X. tropicalis KRSRKNIRSKAEPIGEEDSS.....
TSSC4V2: F201A/N202A/F315A/R316A
TSSC4V3: W162A/Y165A/F201A/N202A/F315A/R316A

Supplementary Figure S4. Multiple sequence alignment of TSSC4 homologs

Multiple sequence alignment of TSSC4 homologs. Species are indicated on the left. *H. sapiens*, *homo sapiens*; *P. troglodytes*, *Pan troglodytes*; *M. mulatta*, *Macaca mulatta*; *C. lupus*, *Canis lupus*; *B. taurus*, *Bos taurus*; *R. norvegicus*, *Rattus norvegicus*; *M. musculus*, *Mus musculus*; *G. gallus*, *Gallus gallus*; *X. tropicalis*, *Xenopus tropicalis*. Residues are colored light to dark red according to increasing level of conservation. Residue numbering according to human TSSC4 is indicated above the alignment. $PRPF8^{Jab1\Delta C}$-binding and $SNRNP200^{HR}$-binding regions (regions 1-4) are indicated by black lines above the alignment. Residues exchanged for alanines to generate the non-binding $TSSC4^{V3}$ are highlighted by cyan background. The alignment was prepared with Homologene (NCBI) employing Clustal Omega (2) and shaded with ALSCRIPT (3).

SUPPLEMENTARY FILES

Supplementary File S1. Structural comparison to the U4/U6-U5 tri-snRNP

PyMOL session file of the structural comparison shown in Figure 6A.

SUPPLEMENTARY REFERENCES

1. Williams, C.J., Headd, J.J., Moriarty, N.W., Prisant, M.G., Videau, L.L., Deis, L.N., Verma, V., Keedy, D.A., Hintze, B.J., Chen, V.B. *et al.* (2018) MolProbity: More and better reference data for improved all-atom structure validation. *Protein Sci*, **27**, 293-315.
2. Sievers, F., Wilm, A., Dineen, D., Gibson, T.J., Karplus, K., Li, W., Lopez, R., McWilliam, H., Remmert, M., Soding, J. *et al.* (2011) Fast, scalable generation of high-quality protein multiple sequence alignments using Clustal Omega. *Mol Syst Biol*, **7**, 539.
3. Barton, G.J. (1993) ALSCRIPT: a tool to format multiple sequence alignments. *Protein Eng*, **6**, 37-40.
